# Contextualized sensorimotor norms: Multi-dimensional measures of sensorimotor strength for ambiguous English words, in context

**DOI:** 10.3758/s13428-026-03134-6

**Published:** 2026-08-12

**Authors:** Sean Trott, Benjamin Bergen

**Affiliations:** 1 Rutgers University-Newark, Newark, NJ USA; 2 UC San Diego, La Jolla, CA USA

## Abstract

Embodied theories of language emphasize the role of sensorimotor experience in linguistic knowledge. Central to testing these theories is the creation of large datasets of *linguistic norms*, which contain judgments about a word’s sensorimotor associations and can be used to predict human behavioral or brain data – sometimes in contrast to competing variables, such as those derived from distributional language models. Yet many of these datasets contain judgments about words in isolation, despite the fact that most words are ambiguous, making it difficult to determine *which meaning* of a word is characterized by its rating (e.g., “wooden table” vs. “data table”). In the current work, we introduce a new lexical resource (directly inspired by the Lancaster sensorimotor for 112 English words, each rated in four different contexts (448 sentences total). We demonstrate: first, that these ratings encode overlapping but distinct information from the Lancaster sensorimotor norms; second, that decontextualized ratings likely reflect the more *dominant* meaning of ambiguous words; third, that homonyms have more distinct sensorimotor profiles than polysemes; fourth, that the *contextualized sensorimotor distance* between two uses of an ambiguous word predicts human judgments about semantic relatedness; and fifth, that ratings derived from GPT-4 align reasonably well with human judgments. We conclude by suggesting that contextualized ratings like these can be used both to inform competing theories of semantic representations and also to evaluate or “probe” the ability of LMs to recover sensorimotor information.

## Introduction

How do humans represent the meaning of words? Many theories suggest that humans *ground* the word meaning in a rich network of sensorimotor associations (Pulvermüller, 1999; Bergen, 2012; Bergen & Feldman, 2008; Barsalou, 1999; Winter & Bergen, 2012; Barsalou, 2008; Glenberg & Kaschak, 2002). These theories are typically contrasted with *amodal* theories of semantics, in which word meanings are represented as abstract symbols (Fodor, 1975); or with *distributional* theories, in which word meanings are derived from the linguistic contexts in which those words occur (Harris, 1954; Lenci, 2018). For instance, embodied theories might argue that our representation of the word “table” incorporates not just the words that “table” frequently co-occurs with, but also our experience with tables in the physical world: how they look and feel, how our bodies interact with them, and more.

Crucial to testing these theories is the development of large-scale *norming databases*, such as the Lancaster sensorimotor (LS) norms (Lynott, Connell, Brysbaert, Brand, & Carney, 2019; Wingfield & Connell, 2023). The LS norms provide human ratings of approximately 40,000 English words across six sensory domains (e.g., vision or touch) and five motor domains (e.g., foot/leg or hand/arm). These norms – and others like them – have been used to predict human behavioral and neural responses to words (Connell & Lynott, 2012; Lynott et al., 2019; Vinaya, Trott, Pecher, Zeelenberg, & Coulson, 2025), and there is growing evidence for the hypothesis that representations of word meaning are informed by sensorimotor experience.

Despite the promise and early success of this approach, it faces a key limitation: with some exceptions (Scott, Keitel, Becirspahic, Yao, & Sereno, 2019), resources like the LS Norms typically contain judgments about the meaning of a word in isolation. In practice, however, many words are *ambiguous*, i.e., they mean different things in different contexts (Haber & Poesio, 2021; Rodd, Gaskell, & Marslen-Wilson, 2004). In English, anywhere from 7% (Rodd et al., 2004) to 15% (Trott & Bergen, 2020) of words have multiple, unrelated meanings—and as many as 84% are polysemous, having multiple, related meanings (Rodd et al., 2004). For example, “table” may refer to furniture, a database organized into rows and columns, or even the act of postponing a meeting. Further, even in its furniture sense, different uses of “table” may evoke different sensorimotor associations (e.g., “She polished the table” vs. “She lifted the table”) (Elman, 2009; Trott, Torrent, Chang, & Schneider, 2020; Yee & Thompson-Schill, 2016). Indeed, ratings of sensorimotor strength or concreteness demonstrably vary depending on which context a word is presented in Reijnierse, Burgers, Bolognesi, and Krennmayr (2019) and Scott et al. (2019). Accordingly, context-sensitivity plays an important role in theories of flexible semantic representation (Elman, 2009; Yee & Thompson-Schill, 2016; Trott & Bergen, 2023), but ratings of words in isolation struggle to capture that flexibility.

This limitation also connects to an ongoing debate about how best to build computational models of language. Systems like transformer language models (LMs) display remarkable performance across a variety of domains, but have been criticized for a lack of sensorimotor grounding (Harnad, 1990; Bender & Koller, 2020; Bisk et al., 2020; Tamari et al., 2020). One response has been to use sensorimotor norms to either augment the training of these models (Kennington, 2021; Wan, Kedzie, Ladhak, Carpuat, & McKeown, 2020a; Wan, Xing, Su, Liu, & Huang, 2020b) or probe the degree to which their word representations *reflect* sensorimotor information (even if the models themselves lack in embodied experience) (Utsumi, 2020; Fernandino, Tong, Conant, Humphries, & Binder, 2022; Marjieh, Sucholutsky, van Rijn, Jacoby, & Griffiths, 2024). Notably, even LMs trained on linguistic input alone display a surprising degree of social and physical knowledge (Jones et al., 2022; Jones & Bergen, 2024; Marjieh et al., 2024; Trott, Jones, Chang, Michaelov, & Bergen, 2023), though they typically under-perform humans on the same tasks. This suggests that purely distributional information may play an important role in human semantic and conceptual knowledge, providing support for *hybrid theories* that emphasize both distributional and sensorimotor information (Andrews, Vigliocco, & Vinson, 2009; Andrews, Frank, & Vigliocco, 2014; Davis & Yee, 2021; Vinaya et al., 2025).

Again, however, testing such theories requires carefully measuring how sensorimotor associations vary not only *across words* but *across contexts* for the same word. In the present work, we introduce the contextualized sensorimotor norms (CS norms), a dataset of sensorimotor judgments for ambiguous words in minimal pair sentential contexts. Our primary goal in the current work is to demonstrate the utility of such a dataset for even a relatively small sample of words and contexts, i.e., illustrating that sensorimotor associations do reliably vary across contexts, that such variance can be used to test theoretical questions about the nature of semantic representations, and that such a dataset can be used to validate automated approaches to scaling and generalization.

In Section “Contextualized sensorimotor norms”, we describe the creation of the dataset and, following Lynott et al. (2019), provide descriptive statistics about the strength and correlation between each sensorimotor dimension. We then quantify the distribution and degree of contextual variance in sensorimotor associations in several ways, including deviations from the original Lancaster sensorimotor norms (Section “Deviations from the Lancaster sensorimotor norms”), the range of sensorimotor associations across contexts for a word (Section “Within-word sensorimotor variance”), and the relationship between meaning dominance and sensorimotor strength (Section “Dominance and sensorimotor strength”). We find that contextualized sensorimotor strength covaries with both *meaning dominance* and similarity to decontextualized ratings of the same word – suggesting that ratings of ambiguous words in isolation likely capture the more dominant (and more concrete) meaning of those words (e.g., “wooden table” vs. “data table”). Section “Comparing distributional and sensorimotor similarity” then illustrates the utility of the CS norms in informing psycholinguistic theory by demonstrating that a metric derived from the norms – the sensorimotor distance between contexts of use – improves predictions of human relatedness judgments about ambiguous words, above and beyond a similar metric derived from the distributional statistics of word usage (Vinaya et al., 2025). In Section “Case study: Can large language models help scale the CS norms?”, we test the viability of scaling the CS norms with GPT-4, a large language model (LLM). Finally, Section “General discussion” discusses limitations of these norms and avenues for future research.

### Related work

#### Related resources

A number of existing resources provide information about word concreteness or imageability (Brysbaert, Warriner, & Kuperman, 2014b; Coltheart, 1981). For example, the Brysbaert concreteness norms contain concreteness judgments for approximately 37,000 English words (Brysbaert et al., 2014b); concreteness ratings have also been collected for Dutch (Brysbaert, Stevens, De Deyne, Voorspoels, & Storms, 2014a), Croatian (Ćoso, Guasch, Ferré, & Hinojosa, 2019), and more.

One disadvantage of judgments of overall concreteness or imageability is that they do not differentiate by sensorimotor feature. As others have reported (Connell & Lynott, 2012, 2016), these judgments may overrepresent particular sensory modalities (such as vision) and underrepresent others (such as olfaction). These systematic differences in which dimensions are most reflected in aggregate judgments may account for the fact that such norms are less predictive of measures of online processing (e.g., in a lexical decision task) than measures derived from modality-specific judgments (Connell & Lynott, 2016). Beyond differences in their predictive power, modality-specific norms are also simply more informative about the kinds of grounded information associated with a particular word. As Lynott et al. (2019) point out, disentangling sensorimotor dimensions can be helpful for determining which aspects of sensorimotor experience are most relevant to certain kinds of semantic judgments or lexical processing.

More recently, researchers have collected ratings about multiple semantic features for each word, including sensorimotor associations (Lynott et al., 2019), as well as more fine-grained judgments within each modality (e.g., for Vision, whether the referent is Fast or Slow; for Touch, whether it is Hot or Cold) (Binder et al., 2016). Of these, the largest dataset is the Lancaster sensorimotor norms (Lynott et al., 2019), which includes 11-dimensional judgments for about 40,000 English words. This approach has been extended to French (Miceli, Wauthia, Lefebvre, Ris, & Simoes Loureiro, 2021) and Dutch (Speed & Brybaert, 2021). In each case, the words were presented without context. Several related datasets include judgments about *body-object-interaction (BOI)*, i.e., the ease with which the human body can interact with the referent of a word (Muraki, Siddiqui, & Pexman, 2022; Pexman, Muraki, Sidhu, Siakaluk, & Yap, 2019). Finally, several studies collect concreteness judgments about words in context (Scott et al., 2019; Reijnierse et al., 2019). However, to our knowledge, no dataset includes judgments about different sensorimotor features across linguistic contexts.

As discussed earlier, these norming datasets (like the Lancaster sensorimotor norms) have been immensely useful across disciplines. Applications include: investigating lexicon-wide relationships with other variables, such as iconicity (Winter, Lupyan, Perry, Dingemanse, & Perlman, 2024); predicting behavioral and brain responses to lexical stimuli (Lynott et al., 2019; Wingfield & Connell, 2023; Vinaya et al., 2025); studying the trajectory of word learning during development (Campbell, Casillas, & Bergelson, 2024; Muraki et al., 2022; Ponari, Norbury, & Vigliocco, 2020); and even evaluating the ability of language models to recover sensorimotor information (Hagihara & Miyazawa, 2026; Kewenig, Skipper, & Vigliocco, 2025; Xu et al., 2025).

#### Grounding language models in psycholinguistic data

Recent work in natural language processing (NLP) has begun to incorporate these psycholinguistic resources. One approach predicts human judgments about concreteness or salient sensorimotor features from LM representations, with varying degrees of success (Chersoni, Xiang, Lu, & Huang, 2020, Turton, Vinson, & Smith, 2020; Thompson & Lupyan, 2018; Utsumi, 2020). Another approach uses sensorimotor features to augment an LM’s performance on an applied task, such as the GLUE benchmark (Kennington, 2021) or metaphor detection (Wan et al., 2020b). As mentioned in Section “Introduction”, these experiments are limited in that the sensorimotor features themselves were obtained for words in isolation.

#### Grounding language models with multimodal input

An alternative approach is to ground LM representations more directly in multimodal input. Most of this work has emphasized the visual modality, linking sentences to static images (Jia et al., 2021; Kim, Son, & Kim, 2021; Kiros, Chan, & Hinton, 2018; Singh et al., 2022; Schuhmann et al., 2022; Su et al., 2020) or video (Zellers et al., 2021). This paradigm has seen remarkable progress in recent years, with the release of commercially available “multimodal” models like GPT-4o (OpenAI, 2024). Despite this progress, this approach is both time-consuming and costly – and, as in some areas of psychology research, has neglected certain sensory domains such as taste and smell (Speed & Majid, 2020). Nevertheless, it is likely to play a key role in applied (or even theoretical) work on grounding LMs. Yet *norms* based on human judgments about interpretable, psychologically meaningful dimensions of experience are still crucial for investigating psycholinguistic theories – and perhaps even evaluating or probing multimodal LMs (Xu et al., 2025; Hagihara & Miyazawa, 2026; Trott, 2024a).

## Contextualized sensorimotor norms

We collected sensorimotor judgments about ambiguous words, appearing in controlled sentential contexts.

### Materials

We used sentences from the RAW-C (relatedness of ambiguous words–in context) dataset (Trott & Bergen, 2021). RAW-C contains relatedness judgments for 672 English sentence pairs, each containing the same target word (e.g., “bat”) in either the same sense (e.g., “furry bat” vs. “fruit bat”) or different sense (e.g., “furry bat” vs. “wooden bat”); it also contains dominance judgments about the relative salience of each meaning. Same vs. different sense was coded in the original RAW-C dataset and was based on whether the meanings were listed separately in both the Merriam-Webster Dictionary and Oxford English Dictionary; meanings listed as different entries were annotated as homonymy, while meanings listed as different senses under the same entry were annotated as polysemy. Past work (Trott & Bergen, 2021) found that relatedness judgments were significantly lower for contexts coded as different sense. There were 448 unique sentences in total (112 target words, with four sentences each). Participants made judgments about the sensorimotor associations evoked by the target word in each sentence. This provided a direct analog to the Lancaster sensorimotor norms (Lynott et al., 2019).

### Participants

We recruited participants until each sentence had at least ten observations, after applying the exclusion criteria. In all, 377 participants participated through UC San Diego’s Psychology Human Subject Pool. Participants received class credit for participation. After excluding non-native speakers of English, participants who failed to pass the bot checks, and participants whose inter-annotator agreement score was too low (see Section “Inter-annotator agreement”), 283 participants remained. Of these, 223 identified as female (47 male, eight non-binary, and five preferred not to answer). The mean self-reported age was 20.4 (median = 20, SD = 2.98), ranging from 18 to 43.

### Procedure

We adapted the procedure from Lynott et al. (2019), with participants now seeing words in sentential contexts. Participants were randomly assigned to one of two judgment types: 1) perception, rating a word’s associated sensory modalities (vision, hearing, touch, interoception, smell, and taste); and 2) Action, rating its associated action effectors (hand/arm, foot/leg, mouth/throat, head, and torso). In total, 132 participants were assigned to the perception judgment type, and 151 were assigned to the action judgment type.

After consenting, participants answered two bot check questions. They were then told that they would read a series of sentences, each containing a bolded word (e.g., “It was a wooden **table**”), and that their task was to rate the degree to which they experienced the concept denoted by that word with either six sensory modalities (in the perception judgment type) or five action effectors (in the action judgment type). Ratings ranged from 0 (not at all experienced with that sense/effector) to 5 (experienced greatly with that sense/effector).

Each participant rated approximately 60 sentences overall, randomly sampled from the set of 448 sentences. No participant saw the same target word twice. On each trial, the sentence appeared at the top of the page, with the target word bolded. Underneath the sentence, the instructions read: “To what extent do you experience WORD:” (for perception) or “To what extent do you experience WORD by performing an action with the:” (for action), where “WORD” was replaced with the target word. Underneath the instructions were six (for perception) or five (for action) rating scales, corresponding to each possible sensory modality or action effector. For the action judgment type, the scale was accompanied by a labeled diagram of the body, as in Lynott et al. (2019).

To reach the target of ten respondents to each word in both action and perception tasks, we collected data in two stages. In the first stage (group 1), participants were randomly assigned to either the perception or action judgment types, and the sentences they observed were randomly sampled from the set of possible sentences for each word. After collecting responses from 264 participants in this way, some sentences still had fewer than ten observations, simply by chance – as well as many with more. Thus, in the second stage (group 2), participants were assigned a mix of low-n (sentences with fewer than ten ratings) and high-n (sentences with ten or more ratings) items. The goal was to speed data collection; to control for potential differences across groups, we compared their distributions of inter-annotator agreement scores, and found no evidence of different response behavior in the two stages (see Section “Inter-annotator agreement”).

After the main task, participants self-reported gender, age, and native English speaker status.

Data were collected online using JsPsych (De Leeuw, 2015).

### Inter-annotator agreement

We sought to establish the degree to which different participants agreed about their ratings for each sentence, both to characterize the dataset and to exclude participants whose ratings diverged substantively from the rest of the sample. We used a leave-one-out scheme: for each participant, we computed Spearman’s rank correlation between that participant’s responses and the mean ratings for those items from the rest of the sample.

We did this in two stages. We first computed the distribution of agreement scores for the 264 participants in group 1: the participants who saw a truly random selection of sentences. Based on this distribution of inter-annotator agreement scores, we excluded 18 participants with scores more than 2 standard deviations below the mean for that judgment type. Among the final set of 246 participants in this group, the mean inter-annotator rank correlation was 0.47 for action judgments (SD = 0.1) and 0.64 for perception judgments (SD = 0.11).

Then we considered the 39 participants from group 2, who provided ratings for a restricted set of sentences. For each participant in group 2, we compared the ratings for the high-N items to the mean response for those items in group 1. Excluding participants with low inter-annotator agreement left 37 participants in group 2. The mean rank correlation was 0.5 for action judgments (SD = 0.11) and 0.64 for perception judgments (SD = 0.1).

Finally, we combined the inter-annotator agreement scores from both groups and constructed a linear regression with rank correlation as the dependent variable, and main effects of judgment type (action vs. perception) and group (group 1 vs. group 2), as well as their interaction. There was no significant difference in agreement across groups (p>.1), but agreement was significantly higher for perception ratings than action ratings [β=0.17,SE=0.01,p<.001].

### Creating the norms

We averaged across the (at least) ten ratings per sentence per judgment type to produce a mean and standard deviation for each dimension. For example, the sentence “He saw the furry **bat**” contains the mean (and standard deviation) of judgments about the salience of each sensorimotor feature. The contextualized sensorimotor norms (CS norms) provide the mean sensorimotor strength for 11 dimensions (six sensory modalities and five action effectors) for a target word in a given context.^1^

### Characterizing the contextualized sensorimotor norms

**Fig. 1:**
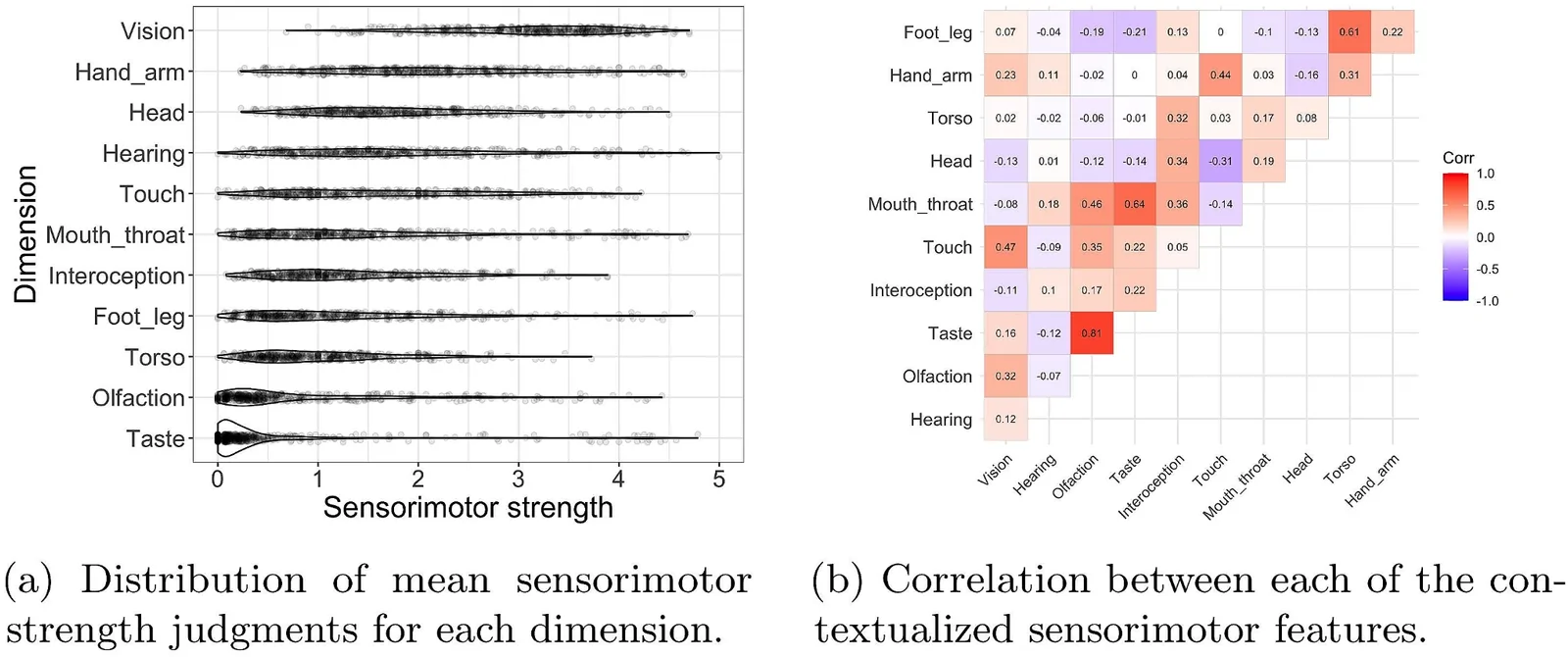
Descriptive statistics for contextualized sensorimotor norms. *Left*: distribution of sensorimotor strength across all contexts for each dimension. *Right*: correlation between dimensions

We first visualized the distribution of judgments for each dimension (see Fig. 1a). Consistent with the original LS norms (Lynott et al., 2019) and work on the English lexicon more generally (Majid, 2020), judgments tended to be highest for the vision dimension and lowest for olfaction and taste. We then asked which dimensions were correlated. Also consistent with past work (Lynott et al., 2019), we found particularly strong positive correlations between olfaction and taste, as well as foot/leg and torso. Additionally, the CS norms had a positive correlation between taste and mouth/throat (see Fig. 1b).

**Fig. 2:**
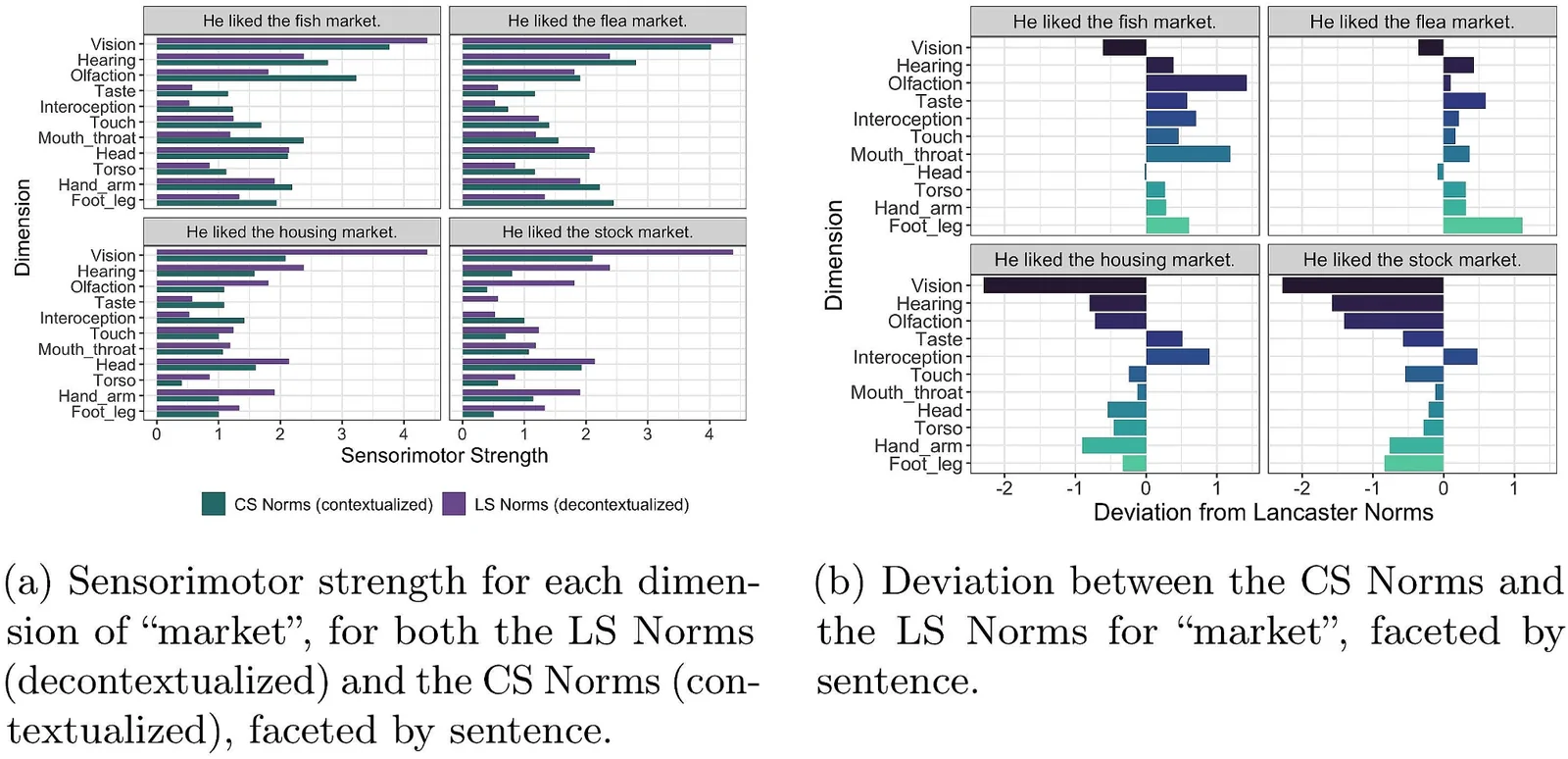
Sensorimotor profiles for the word “market” across four sentential contexts. *Left*: absolute sensorimotor strength for each dimension under the LS norms and the CS norms. *Right*: deviation of the CS norms from the LS norms

## Contextual variance in sensorimotor associations

A key motivation for the CS norms was to account for potential variation within each word in terms of which sensorimotor features were most salient across distinct sentential contexts. To explore and illustrate this variation, we quantified contextual variance in several ways (Sections “Deviations from the Lancaster sensorimotor norms” and “Within-word sensorimotor variance”) and identified systematic sources of variance in which sensorimotor associations were profiled by a given context, including sense-level distinctions (Section “Within-word sensorimotor variance”) and meaning dominance (Section “Dominance and sensorimotor strength”).

### Deviations from the Lancaster sensorimotor norms

First, we normalized the sensorimotor features for each context of use to the mean norms for that word from the LS norms. For example, the LS norms have a single 11-dimensional vector for the word “market”. For each of the four sentential contexts in which “market” appeared, we calculated the difference in mean ratings between our norms and the LS norms. This provides an estimate of the degree to which the human judgments were impacted by the sentential context for each sensorimotor dimension, beyond the word’s meaning in isolation.

Figure 2 shows both the raw LS norms and CS norms for the word “market” (Fig. 2a) and the deviations between the LS norms and CS norms for each sentence (Fig. 2b). The deviations from the LS norms track the two senses of the word being profiled. The two sentences corresponding to the *location* sense of “market” (i.e., “fish market” and “flea market”) were closer to the LS norms (i.e., the deviations were smaller on average); the notable exceptions were the *olfactory* and *mouth/throat* dimensions for the “fish market” context, and the *foot/leg* dimension for the “flea market” context. Both deviations are facially reasonable: a fish market likely connotes sensorimotor representations relating to smell and eating, and a flea market likely connotes representations relating to the experience of moving in space. In contrast, the sentences corresponding to the *financial* sense of “market” (i.e., “housing market” and “stock market”) were considerably lower in sensorimotor strength across almost all dimensions, especially *vision*. This again makes sense, given that this meaning is more metaphorical or abstract than the *location* meaning of “market”; apart from representations of their performance, neither housing markets nor stock markets can be visually perceived in the way that fish markets and flea markets can. See Fig. 11 for an analogous set of plots of several other words with particularly strong contextual variation, including “jam”, “punch”, and “match”.

**Fig. 3:**
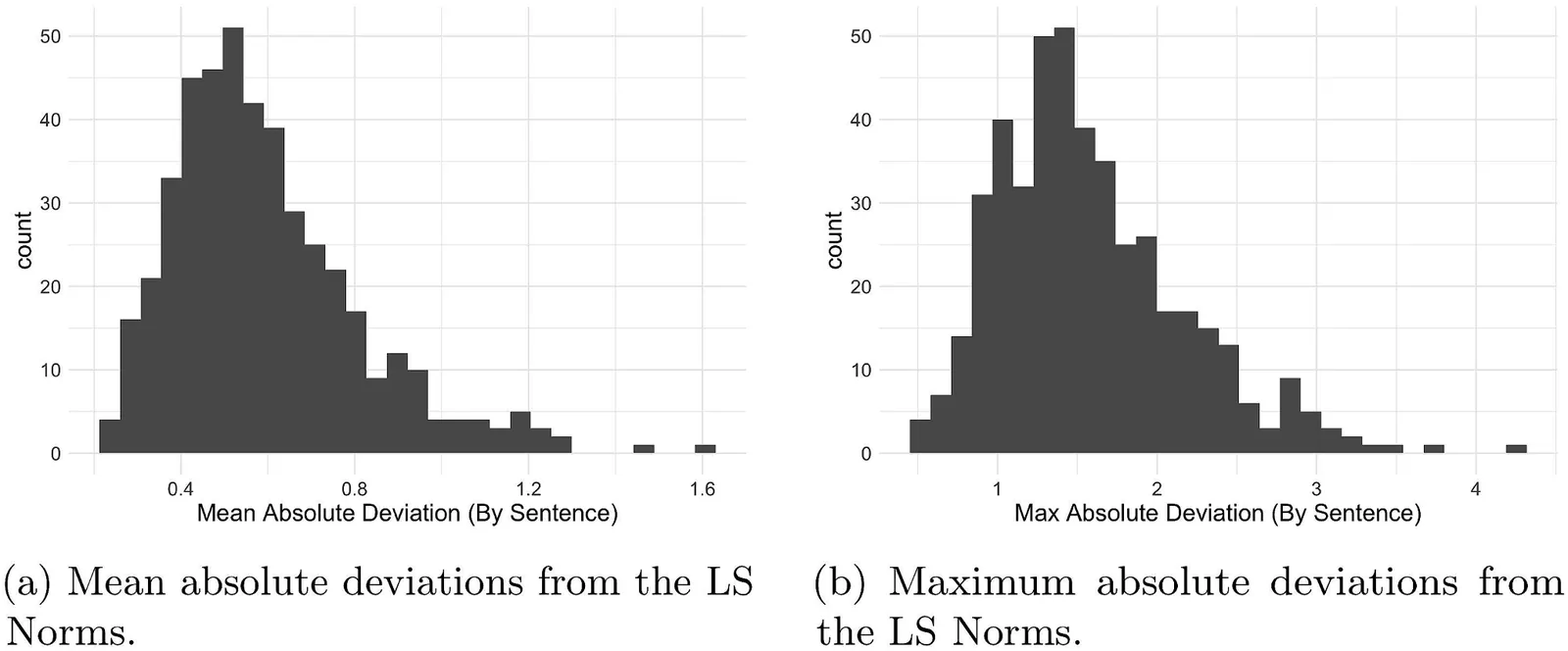
Mean absolute differences (*left*) and maximum absolute differences (*right*) from the LS norms, across sentences

Of course, it is difficult to draw generalizations on the basis of individual examples alone. Are words like “market” the exception, or is contextual variance in sensorimotor associations pervasive across the dataset? One way to address this question is to consider the distribution of *mean absolute contextual deviation* (i.e., from the LS norms) across sentences in the CS norms dataset, as depicted in Fig. 3a. Here, each sentence is associated with a mean absolute deviation score, representing the average (absolute) differences across that sentence’s sensorimotor dimensions and those same dimensions for the word in isolation. The mean absolute sentence-level deviation was 0.59 (SD=0.22), and ranged from 0.23 to 1.6. Another perspective comes from visualizing the distribution of *maximum absolute contextual deviations* (Fig. 3b), i.e., the largest deviation across the 11 sensorimotor dimensions for each sentence context. Both distributions are right-skewed, but the range of maximum deviations is (unsurprisingly) larger, extending up to 4.25 (for *jam* in “It was a traffic jam”; see also Fig. 11a).

**Fig. 4:**
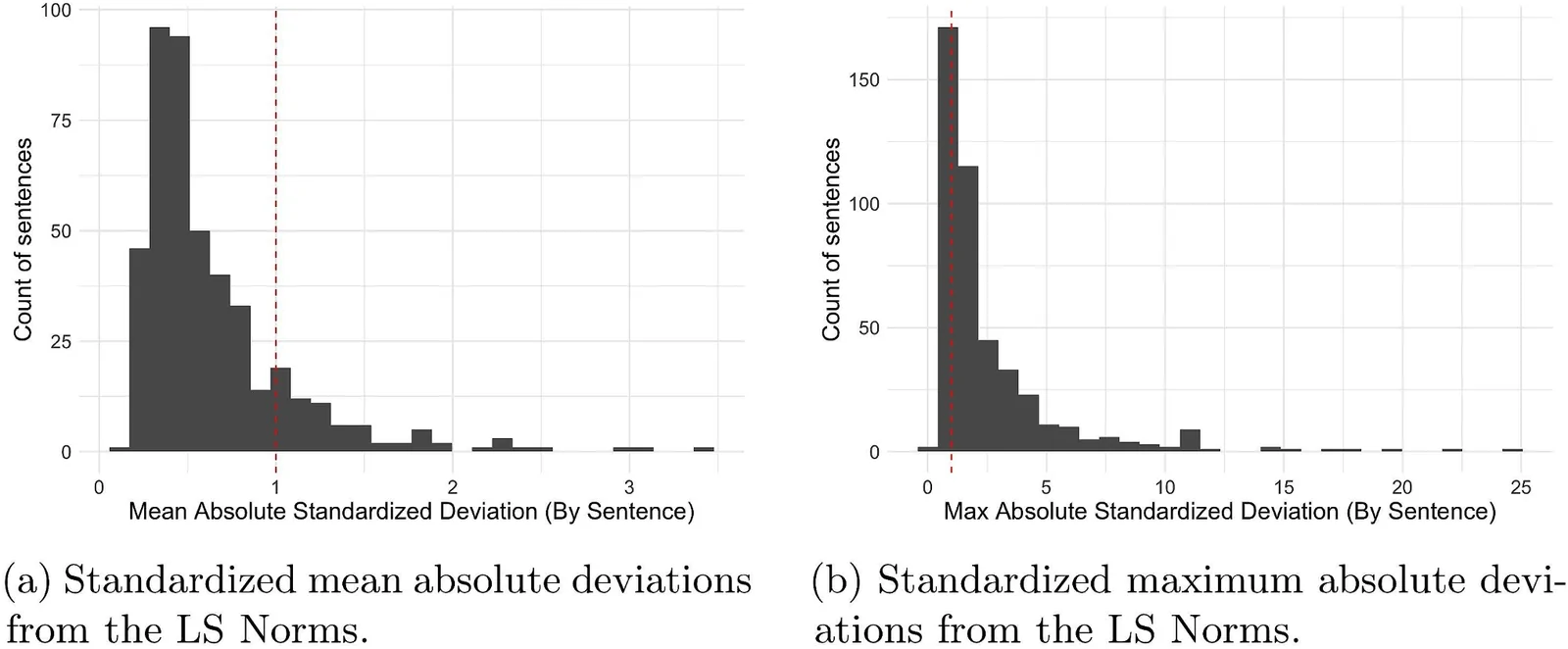
Standardized mean absolute differences (*left*) and maximum absolute differences (*right*) from the LS norms, across sentences. Standardization was implemented by dividing the difference scores in each dimension of a sentential context by the published *SD* for that word in the Lancaster sensorimotor norms

One challenge with interpreting deviations on the original LS norms scale is determining whether a difference of a given magnitude is *meaningful*. For example, a deviation of 0.5 on a 5-point scale could reflect either a substantial shift in sensorimotor associations across contexts or sampling error – depending on how much variability the original annotators for a word exhibited when providing judgments. To address this, we divided the absolute deviation for each dimension of each sentence (as described above) by the published standard deviation for that dimension of that word in the original LS norms (with a constant of 0.1 added to account for words with 0 SD). This *standardized absolute deviation* measure thus represents the number of standard deviations between the contextualized rating and the LS norm for a given sentence and dimension. Note that for a subset of word x dimension cells in the original LS norm dataset, the standard deviation was 0; these mostly reflected floor effects for the Olfaction and Taste dimensions. Figure 4 shows the resulting distribution of by-sentence standardized deviations, illustrating both the mean across dimensions (Fig. 4a) and the maximum deviation across dimensions (Fig. 4b). The mean standardized deviation across sentences was 0.64 (SD=0.45), and ranged from 0.17 to 3.47. As with the raw absolute deviations, the range is considerably larger when considering the maximally divergent dimension rather than the average deviation across dimensions, i.e., up to 25. These extreme cases are in part attributable to floor effects for certain dimensions in the original LS norms, e.g., cases in which raters unanimously agreed on a low rating for a given word. While the magnitude of the standardized deviation is amplified by the small denominator, these cases are still illustrative: they suggest that linguistic context can substantively shift the sensorimotor associations for an ambiguous word relative to the associations accessed for that word in isolation – perhaps reflecting (as discussed in Section “Sense dominance and deviation from the Lancaster norms” below) systematic differences in which meaning comes to mind more easily.

The standardized and non-standardized measures of sentence-level mean absolute deviation from the LS norms exhibit a moderately strong positive correlation (r=0.44,p<0.001, ρ=0.55,p<0.001), though (in large part because of the floor effects mentioned above) they are by no means identical. In our view, they address different questions: the raw deviations reflect shifts in terms of the original rating units, while the standardized deviations account for the degree of variability among the LS Norm annotators for a given word.

### Within-word sensorimotor variance

Above, we found that a number of sentences exhibited different sensorimotor profiles than ratings of ambiguous words in isolation. Here, we aimed to quantify and characterize the degree of *within-word contextual variance*. That is, independent of deviation from the LS norms, we identified how much the sensorimotor associations of a word shifted across contexts, and whether we could account for systematic variance in which words displayed the most variance. For each word, we quantified two sources of variance: the *between-sense* range (i.e., the extent to which sensorimotor associations differed between the two senses of a word), and the *within-sense* range (i.e., the extent to which sensorimotor associations differed between two contexts of the same sense). In each case, we analyzed both the mean and maximum range: the former captures the average degree of divergence across all 11 dimensions, while the latter captures the degree of divergence on the most distinct dimension.

First, we asked whether the range of sensorimotor associations was larger within or between senses across words in the dataset. For both the mean and maximum range measures, we fit a linear mixed model predicting range from a factor representing same vs. different sense. Same sense contexts were associated with a significantly smaller mean range [β=-0.13,SE=0.02,p<0.001] and maximum range [β=-0.58,SE=0.07,p<0.001]. That is, a word’s sensorimotor associations were more similar for contexts conveying the same sense than for those conveying different senses.

We then asked whether distinct types of ambiguity (polysemy vs. homonymy) were associated with a different degree of sensorimotor variance. Specifically, we predicted that polysemous meanings would exhibit lower sensorimotor variance than homonymous meanings, given that polysemous meanings are more related on average (e.g., “marinated lamb” vs. “friendly lamb”) than homonymous ones (e.g., “wooden bat” vs. “furry bat”). For both range measures, we fit a linear mixed model predicting range, with fixed effects of ambiguity type (polysemy vs. homonymy) and sense boundary (same sense vs. different sense), an interaction between those factors, and random intercepts for word. Table 1 depicts the coefficients and standard errors for each of the model fits; note that the reference level (i.e., the intercept) corresponds to between-sense homonyms, so other coefficients should be interpreted in reference to this. With that in mind, we identified significant fixed effects of both ambiguity type and sense boundary for both the mean and max range measures: within-sense ranges were smaller than between-sense ranges, and polysemes exhibited smaller between-sense ranges than homonyms. Crucially, the interaction between the two factors was significant when predicting max range, and bordered on significant when predicting mean range: that is, the gap between homonyms and polysemes was larger for between-sense than within-sense comparisons. Descriptively, homonyms diverged by an average of 1.79 on their most divergent dimension, while polysemes differed by an average of 1.37; in contrast, within-sense divergence was virtually identical across ambiguity types (0.94 vs. 0.92).

**Table 1:** Coefficient estimates (β) and standard errors from linear mixed-effects models predicting within-word range of CS ratings from ambiguity type (homonymy vs. polysemy), comparison type (different sense vs. same sense), and their interaction, with random intercepts by word. Reference levels are homonymy and different sense, respectively. Significance levels: * p<.05, ** p<.01, *** p<.001

	Mean range	Max range
Intercept (Homonymy, different sense)	0.64 (0.03)***	1.79 (0.08)***
Polysemy	-0.10 (0.04)**	-0.43 (0.09)***
Same sense	-0.18 (0.04)***	-0.85 (0.11)***
Polysemy × Same sense	0.09 (0.05)	0.41 (0.13)**

Together, these results point to two conclusions. First, the contexts conveying distinct meanings of an ambiguous word have more divergent sensorimotor associations than contexts conveying the same thing, and second, homonyms are associated with more distinct sensorimotor profiles than polysemes. Note that both results are also consistent with the findings reported in Section “Do different senses have more distinct sensorimotor profiles?”, where we replicate the same pattern using *sensorimotor distance* between sentence pairs as an alternative operationalization of contextual variance. One practical conclusion of this result is that homonymous senses may deserve particularly high priority when collecting context-specific sensory ratings, given that their distinct meanings have more distinct sensorimotor associations.

### Sense dominance and deviation from the Lancaster norms

Having established that contexts of use exhibit considerable variance in their sensorimotor associations – both from each other, and from the original LS norms for a given word in isolation – we asked whether there is systematic psychological structure to these deviations. One candidate is *sense dominance*: when LS raters provide a rating for an ambiguous word in isolation, do their judgments about a word’s sensorimotor associations reflect the dominant sense more than the subordinate senses?

Dominance is a well-documented property of ambiguous words, with a large body of evidence that it plays a role in lexical processing, particularly for homonyms. Specifically, empirical evidence suggests that comprehenders often activate the more dominant sense of a homonym, even when the linguistic context supports the subordinate meaning (Binder & Rayner, 1998; Duffy, Morris, & Rayner, 1988; Rayner, Pacht, & Duffy, 1994); this is called the *subordinate-bias effect*. For example, the “data table” sense of *table* might be less dominant than the “wooden table” sense.

As suggested above, the observation that some senses are more dominant than others also raises important questions for the construction of *norming* datasets, especially when those datasets focus on the psychological properties of words in isolation. The first question, then, is practical: to what extent do ratings of ambiguous words in isolation (e.g., their sensorimotor strength) reflect the more *dominant sense* of that word?

We addressed this question in two ways. First, we calculated the cosine distance between the decontextualized LS norm for each word and the sensorimotor norms for that same word in context. We called this distance to Lancaster. Using the *lme4* package (Bates, Mächler, Bolker, & Walker, 2015) in R, we fit a linear mixed effects model predicting distance to Lancaster, with dominance as a fixed effect (and random intercepts for words). This model explained more variance than a model omitting only dominance $[\chi ^2(1)=10.93, p =.001]$. More dominant senses were closer to the decontextualized norm on average [β=-0.01,SE=0.003,p=.001], consistent with the prediction that the LS norms are more influenced by sensorimotor properties of the dominant sense than those of the subordinate sense.

Second, do more dominant senses have stronger or weaker sensorimotor ratings, on average, than the decontextualized rating for that word? For each sentential context, we computed the average difference between each dimension and the corresponding LS norms, such that a positive value reflects a *more* concrete context. A model predicting this measure was significantly improved by the addition of dominance $[\chi ^2(1)=38.24, p <.001]$. Dominant senses had stronger sensorimotor ratings, on average, than the decontextualized ratings for that same word [β=0.09,SE=0.01,p=.001].

**Fig. 5:**
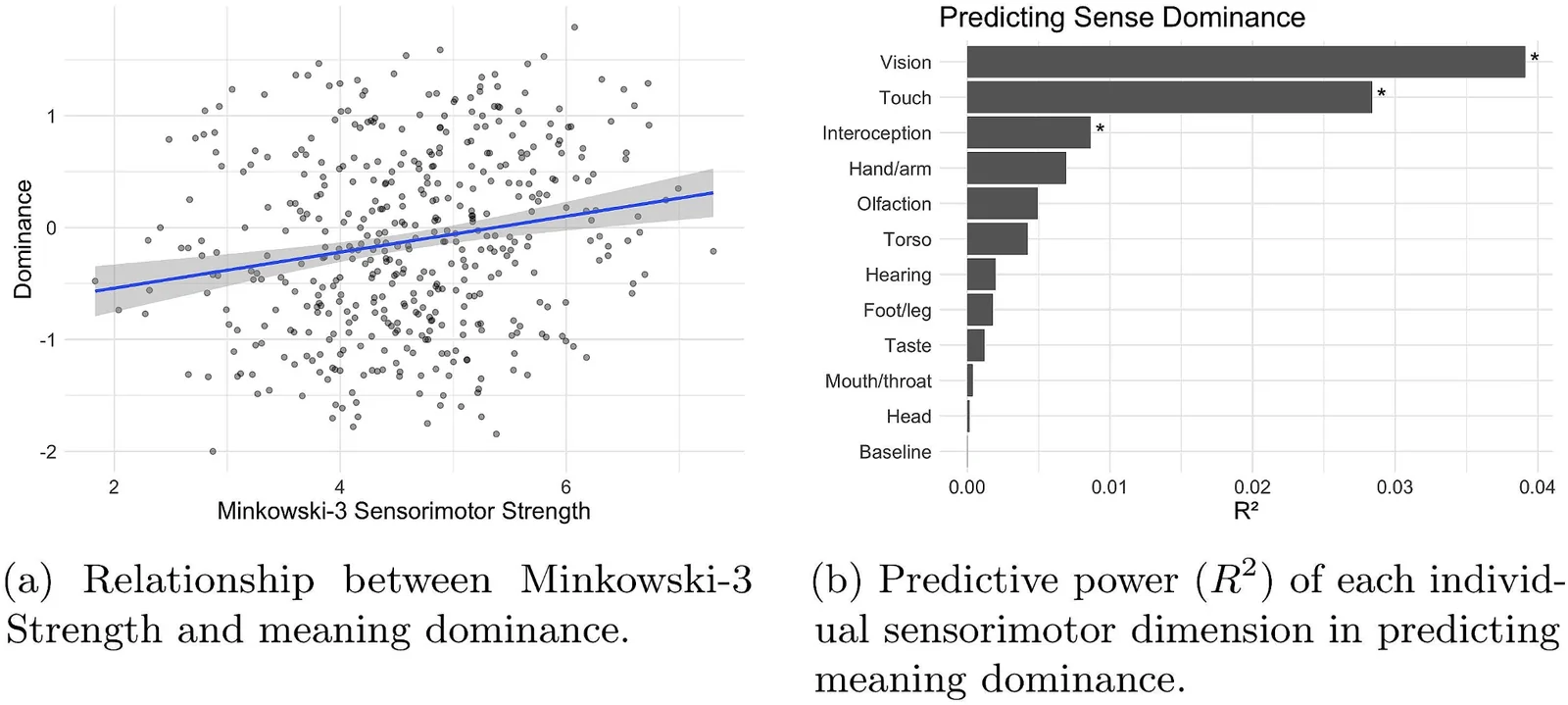
Contextualized meanings with stronger sensorimotor associations overall were also independently judged to be more dominant (*left*). The specific dimensions most useful for predicting dominance were vision, touch, and interoception

Together, these results point to two practical conclusions: first, ratings of the sensorimotor properties of ambiguous words in isolation are likely *more* influenced by the more dominant sense of those words; and second, more subordinate senses tend to be *lower* in sensorimotor strength overall than the rating for the decontextualized word.

### Dominance and sensorimotor strength

Previous work also suggests that more dominant meanings tend to be more *concrete* (Gilhooly & Logie, 1980). As discussed earlier, the construct of concreteness bears considerable similarities to sensorimotor strength – with a key difference being that sensorimotor strength was assessed across different modalities and effectors individually (Lynott et al., 2019). Using the CS norms, we asked whether sensorimotor strength covaried with meaning dominance.

First, following Lynott et al. (2019), we created a composite variable called contextualized sensorimotor strength, which reflected the degree to which a meaning evoked strong sensorimotor associations overall. We measured this in two ways, both inspired by the analyses in Lynott et al. (2019): maximum strength across dimensions (i.e., the strength for the strongest dimension of a contextualized meaning); and Minkowski-3 (i.e., the cube root of the sum of cubed strength values across all 11 dimensions).^2^ These metrics were positively correlated with each other (r=0.87,p<0.001). Examples of sentences with high sensorimotor strength include “They liked the roasted *chicken*” and “She *ran* the marathon”; examples of sentences with low sensorimotor strength include “It was a criminal *case*” and “They *drained* the budget”.

Then, for both metrics, we built a linear mixed effects model with dominance as a dependent variable, fixed effects of contextualized sensorimotor strength, random intercepts for each word, and two covariates reflecting the decontextualized sensorimotor strength for each *word* (i.e., from the Lancaster sensorimotor norms dataset). The full model explained significantly more variance than the same model omitting only contextualized sensorimotor strength, regardless of whether we used maximum strength $[{\chi }{^2}(1)=18.38, p <.001]$ or Minkowski-3 strength $[{\chi }{^2}(1)=32.32, p <.001]$. Consistent with past work (Gilhooly & Logie, 1980), more dominant contexts of use were associated with a higher maximum strength [β=0.26,SE=0.06,p<.001] and higher Minkowski-3 strength [β=0.28,SE=0.05,p<.001]. See Fig. 5a for an illustration of the relationship between Minkowski-3 strength and dominance.

We then asked whether specific sensorimotor dimensions (and which) predicted sense dominance. To test this, we built a series of linear regression models predicting dominance from each sensorimotor feature in turn (with decontextualized sensorimotor strength as a covariate in each case). We then calculated and compared the $R^2$ of the resulting models. The most predictive dimensions were Vision ($R^2 = 0.04$) and Touch ($R^2 = 0.03$). In both cases, the correlation was positive: that is, more dominant contexts of use were also higher in visual strength (r=0.19) and haptic strength (r=0.17). See Fig. 5b for a depiction of the $R^2$ for each model.

Of course, these findings do not explain *why* more concrete meanings – or those higher in visual or haptic strength specifically – are more dominant than meanings with less sensorimotor strength. It could be that the dominance of a word sense is directly shaped by the sensorimotor strength of that sense; alternatively, more concrete senses might also be learned earlier (and be more frequent) than more abstract senses, which in turn could shape dominance (Gilhooly & Logie, 1980).

## Comparing distributional and sensorimotor similarity

The above analyses demonstrate that the sensorimotor properties of ambiguous words do indeed vary across contexts of use (Section “Deviations from the Lancaster sensorimotor norms”), often in systematic ways (Section “Sense dominance and deviation from the Lancaster norms”). They also suggest that sensorimotor variance is greater for different sense usages than same sense usages, and for homonyms than polysemes (Section “Within-word sensorimotor variance”). Here, we replicate and extend those analyses, asking whether sensorimotor properties predict the relationship *between* contexts of use – i.e., whether distinct senses of a word also have more distinct *sensorimotor profiles*, and whether the *sensorimotor distance* (Wingfield & Connell, 2021) between contexts of use is predictive of human relatedness judgments, as well as measures of online sentence processing. These analyses help illustrate the predictive utility of contextualized sensorimotor ratings.

In this section, we also compared the relative utility (and statistical independence) of sensorimotor information to *distributionally-derived* measures of similarity, e.g., using a language model (LM). This allowed us to ask whether the CS norms captured semantically relevant information that LMs do not, informing questions about the degree to which human semantic knowledge integrates both sensorimotor and distributional information (Andrews et al., 2009, 2014; Louwerse, 2018; Vinaya et al., 2025).

### Methods

#### Calculating sensorimotor distance

We first calculated the cosine distance – referred to here as the sensorimotor distance – between the vectors corresponding to each sentence pair for each word (672 sentence pairs total). Larger distances reflect more dissimilar contexts of use, while smaller distances reflect more similar contexts. (Note that this includes comparisons between contexts corresponding to the same sense and those corresponding to different senses.) This procedure follows the approach adopted in Wingfield and Connell (2021).

#### Calculating distributional distance

Following past work (Haber & Poesio, 2020; Riviere, Beatty-Martínez, & Trott, 2025; Rivière & Trott, 2025; Trott & Bergen, 2021, 2023; Vinaya et al., 2025), we operationalized distributional distance as the cosine distance between the contextualized embeddings of a target ambiguous word across minimal pair sentence contexts. We calculated this measure across a variety of language models (LMs) ranging in size and architecture; we focused specifically on *bidirectional* LMs because a significant portion of the RAW-C sentences contained the disambiguating cue *after* the ambiguous word. We used a total of 12 LMs. Model families included ALBERT (Lan et al., 2019), BERT (Devlin, Chang, Lee, & Toutanova, 2018), DistilBERT (Sanh, Debut, Chaumond, & Wolf, 2019), and RoBERTa (Liu et al., 2019); see Appendix A for the full list of all models used.

**Fig. 6:**
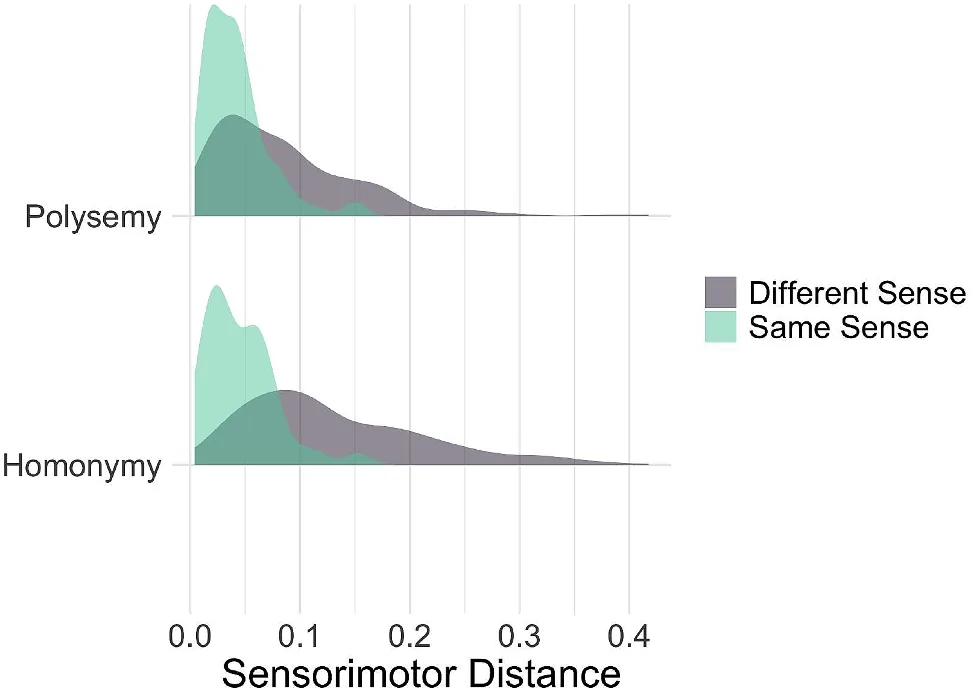
Distribution of sensorimotor distances as a function of same/different sense, as well as the type of ambiguity. Same sense uses had more similar sensorimotor associations than different sense uses

### Sensorimotor distance and word senses

#### Do different senses have more distinct sensorimotor profiles?

Earlier, we found that different-sense usages of an ambiguous word had more distinct sensorimotor profiles, and that homonyms exhibited larger divergences between their most distinct sensorimotor dimension than polysemes (Section “Within-word sensorimotor variance”). Here, we extended this analysis to ask whether it was robust to an alternative operationalization of sensorimotor variance across contexts (i.e., sensorimotor distance).

Indeed, across the entire dataset, different sense contexts appeared to have higher sensorimotor distance than same sense contexts (see Fig. 6), i.e., the distribution of sensorimotor features was more different for different meanings. A mixed effects model predicting sensorimotor distance from sense boundary (different sense vs. same sense), as well as random intercepts for each word, explained significantly more variance than a model omitting only the fixed effect of sense boundary $[\chi ^2 = 67.65, p < 0.001]$.

We then asked whether the effect of sense boundaries was larger for homonyms than polysemes, as suggested by the analysis of within-word ranges in Section “Within-word sensorimotor variance”. To test this, we fit a linear mixed-effects model predicting sensorimotor distance from sense boundary, ambiguity type, and their interaction, with by-word random slopes for the effect of sense boundary, as well as random intercepts for word. This model explained significantly more variance than a model omitting only the interaction $[\chi ^2(1) = 11.54, p < 0.001]$. The coefficients for the model can be interpreted against the reference level (the intercept), corresponding to different sense homonyms, for which the model estimated a sensorimotor distance of 0.13. Same sense homonym pairs had significantly smaller sensorimotor distance [β=-0.08,SE=0.01,p<0.001], as did different sense polysemous pairs [β=-0.05,SE=0.01,p<0.001]. Crucially, the interaction term was significant and positive [β=0.04,SE=0.01,p<0.001], indicating that the gap between homonyms and polysemes was significantly smaller for same sense pairs than different sense pairs.

Concretely: for context pairs conveying different meanings, homonyms had even higher sensorimotor distance (M=0.13,SD=0.08) than polysemes (M=0.08,SD=0.07); in contrast, for context pairs conveying the same meaning, both kinds of ambiguity had virtually identical sensorimotor distance on average (M=0.046 for homonymy and M=0.042 for polysemy). These results are consistent with the findings reported in Section “Within-word sensorimotor variance” using a different measure of contextual variance in sensorimotor associations.

**Fig. 7:**
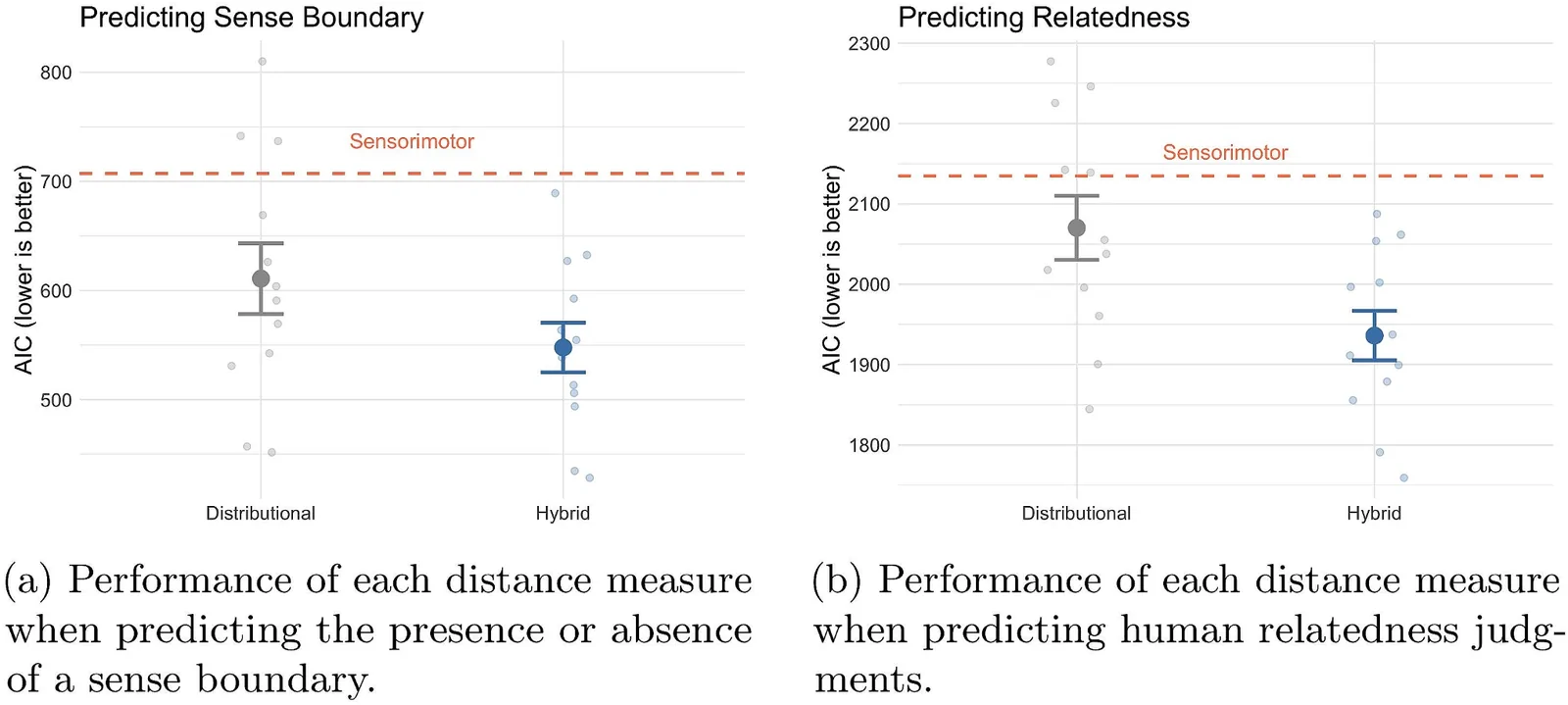
AIC from a regression model containing either distributional distance from a given LM alone, or the combination of distributional and sensorimotor distance. AIC for a model containing only sensorimotor distance depicted as the *orange dashed line*. In all cases, a hybrid model containing both sensorimotor and distributional distance outperformed the regression models with a single distance measure

#### Evaluating sensorimotor vs. distributional distance

We then compared the utility of sensorimotor distance and distributional distance in predicting the presence of a sense boundary. Specifically, we fit a series of logistic regression classifiers predicting sense boundary (a binary variable) using either sensorimotor distance or distributional distance from a given layer of a given LM (see Section “Calculating distributional distance”). We also evaluated a “hybrid” model in each case, i.e., using both sensorimotor distance and distributional distance.

As an evaluation metric for each model, we used Akaike information criterion (AIC), which penalizes the addition of unnecessary parameters. Here, a lower AIC reflects a better model fit; following past work (Burnham & Anderson, 2004; Vinaya et al., 2025), we interpret a difference in AIC of more than 4 (ΔAIC>4) as support for the model with lower AIC. We calculated two ΔAIC measures for each LM: the difference between that LM’s AIC and sensorimotor distance alone ($\Delta AIC_{SM}$) and the difference between that LM’s AIC and the *hybrid* model containing both distance measures ($\Delta AIC_{hybrid}$). In each case, a positive AIC indicates that distributional distance alone was a *worse* predictor than the comparison model: either sensorimotor distance alone ($\Delta AIC_{SM}$) or a model with both predictors ($\Delta AIC_{hybrid}$). All measures were calculated for all layers from each LM, but we report the results only for the *best-performing layer* from each LM.

First, we asked about the direct comparison between distributional distance and sensorimotor distance ($\Delta AIC_{SM}$). Here, $\Delta AIC_{SM}$ was negative in 9 of the 12 LMs tested, i.e., distributional distance alone produced a *better fit* than sensorimotor distance alone. Intriguingly, two of the LMs that performed *worse* than the sensorimotor baseline were multilingual, consistent with past work suggesting that multilingual LMs may suffer from capacity constraints (Chang, Arnett, Tu, & Bergen, 2024), which in turn could affect disambiguation performance in particular (Riviere et al., 2025). See Appendix C for the raw AIC values for each model.

We then asked whether sensorimotor distance provided additional, independent explanatory power above and beyond distributional distance. Specifically, we asked whether a regression model with both predictors exhibited lower AIC than a model with only distributional distance ($\Delta AIC_{hybrid}$). We found that $\Delta AIC_{hybrid} > 4$ for all LMs: that is, in each case, distributional distance alone performed *worse* (higher AIC) than a hybrid model containing both predictors (lower AIC). Note that this was also true for the inverse comparison: each hybrid model was also better than the model with sensorimotor distance alone. See Fig. 7a for an illustration of these findings.

Together, these results suggest that the presence of a sense boundary between two contexts of use (e.g., “fish market” vs. “stock market”) is best modeled using a combination of distributional and sensorimotor information, illustrating the predictive value of the contextualized sensorimotor norms.

### Sensorimotor distance and graded relatedness

Beyond the categorical existence of a sense boundary, a word’s contexts of use also vary in a more graded fashion (Trott & Bergen, 2023; Li & Joanisse, 2021). This is clearest when it comes to different forms of polysemy: “marinated lamb” and “friendly lamb” are different meanings, but are more closely related than “brain cell” and “prison cell”. Using human relatedness judgments from RAW-C (Trott & Bergen, 2021), we asked whether sensorimotor distance reliably predicted the relatedness between two usage contexts for a word – and, as above, whether sensorimotor and distributional information accounted for unique variance in these relatedness judgments.

#### Does sensorimotor distance predict relatedness?

We first calculated the correlation between sensorimotor distance and relatedness. This correlation was significantly negative (r=-0.53,p<0.001), suggesting that contexts with more distinct sensorimotor profiles were judged as less related than contexts with more similar sensorimotor profiles.

#### Evaluating sensorimotor vs. distributional distance

As in Section “Sensorimotor distance and word senses”, we then compared the *predictive power* of sensorimotor distance to distributional distance, as well as a hybrid model containing both distance measures. For each layer of each LM, we calculated the *AIC* of a regression model predicting relatedness from distributional distance; we also calculated the *AIC* for a model containing both distributional and sensorimotor distance. As before, we calculated both $\Delta AIC_{SM}$ ($AIC_{D} - AIC_{SM}$) and $\Delta AIC_{hybrid}$ ($AIC_{D} - AIC_{hybrid}$). In each case, a positive ΔAIC reflects worse performance by distributional distance alone, either compared to sensorimotor distance or to both predictors combined. The results below are reported only for the best-performing layers of each LM.

Considering just the direct comparison between distributional and sensorimotor distance ($\Delta AIC_{SM}$), we found support for distributional distance over sensorimotor distance in seven of the 12 LMs tested; as before, the two multilingual LMs exhibited *worse* fit than sensorimotor distance. We then asked whether the hybrid models improved upon either distributional or sensorimotor distance alone. Crucially, for all LMs tested, we found that they did ($\Delta AIC_{hybrid}>4$ for all LMs tested): a combination of distributional and sensorimotor information was best for predicting human relatedness judgments (see also Fig. 7b and Appendix C).

As with the sense boundary results (Section “Sensorimotor distance and word senses”), these findings suggest that human semantic representations integrate information both about the sensorimotor properties of a word and its distributional profile; this question is also explored in greater detail in the Discussion below.

#### Comparison to LS norms

As a final validation check for the utility of collecting *contextualized ratings* in particular, we constructed a baseline “bag-of-words” model using the decontextualized LS norms. We first identified the disambiguating word across each pair of sentence contexts (e.g., “*furry* bat” vs. “*wooden* bat”); then we calculated the cosine distance between the decontextualized LS norms corresponding to each of those words (e.g., between “furry” and “wooden”). We called this measure the decontextualized distance. We excluded pairs containing disambiguating words that were not included in the LS norms (e.g., “double-sided”), leaving 576 pairs.

A linear mixed effects model including both contextualized sensorimotor distance and decontextualized distance explained more variance than a model omitting only contextualized sensorimotor distance $[\chi ^2(1)=142.07, p <.001]$. This indicates that the CS norms measure encodes additional information beyond what can be inferred by simply identifying the decontextualized sensorimotor properties of other words in the context. This is particularly notable given that the sentences in question were minimal pairs with identical grammatical structure; in circumstances requiring more complex composition operations between the disambiguating words, a bag-of-words approach might be even less effective.

**Fig. 8:**
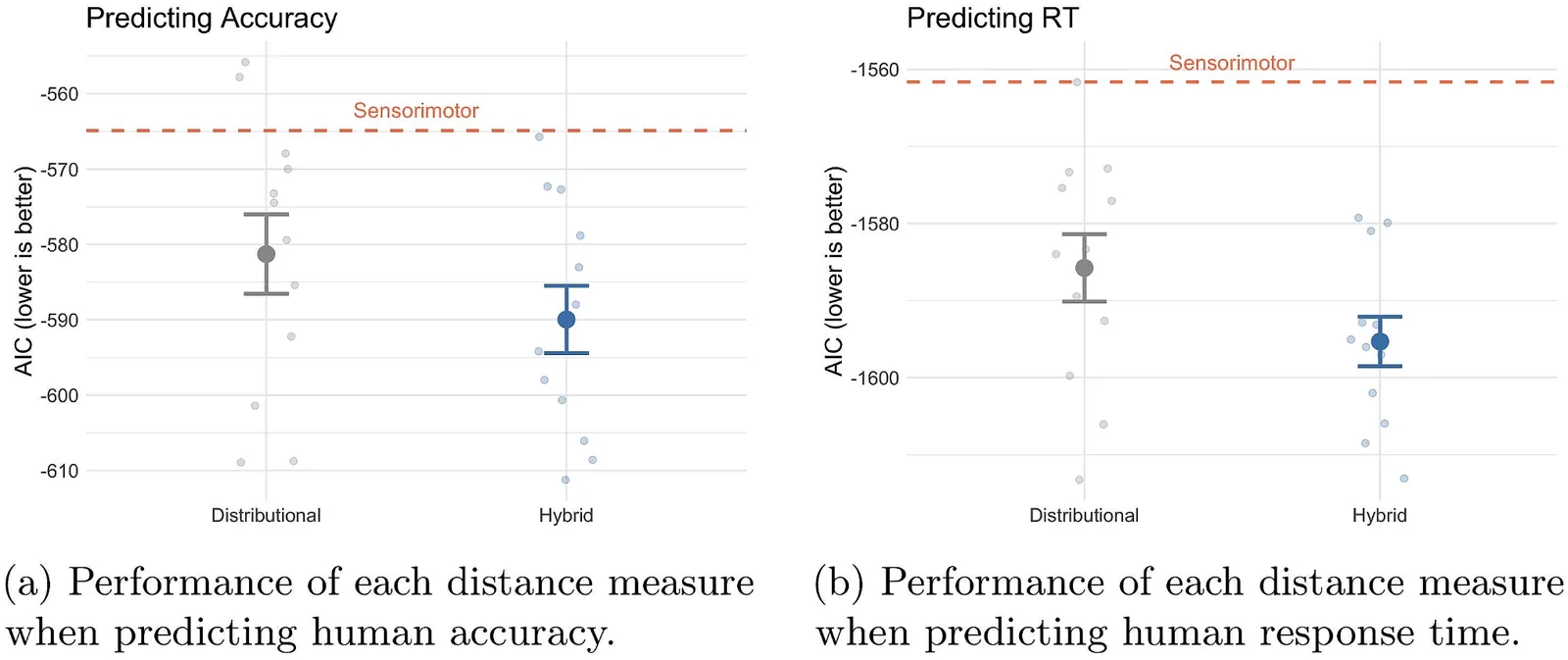
AIC from a regression model containing either distributional distance from a given LM alone, or the combination of distributional and sensorimotor distance. AIC for a model containing only sensorimotor distance depicted as the *orange dashed line*. A hybrid model often out-performed either distance metric alone

### Sensorimotor distance and ambiguity processing

Previous work using a primed sensibility judgment paradigm (Trott & Bergen, 2023) found that human dependent measures (i.e., response time and accuracy) were best predicted by a combination of *categorical* factors (i.e., whether the prime and target sentence conveyed different meanings) and *continuous factors* (i.e., the cosine distance between contextualized representations of an ambiguous word across the prime and target sentence). Crucially, this work relied on distributionally-derived measures of continuous similarity between contexts; one open question is therefore whether the sensorimotor distance between contexts accounts for variance previously attributed to categorical factors.

Here, we extended this work using the CS norms to ask several questions. First, does sensorimotor distance reliably predict measures of online processing for ambiguous words? Second, are these dependent measures best predicted by sensorimotor distance, distributional distance, or (as in Section “Sensorimotor distance and word senses” and “Sensorimotor distance and graded relatedness”) a “hybrid” model accounting for both factors? Third, do both continuous measures together “explain away” the categorical effect of sense boundaries?

#### Explanation of experimental materials

We used publicly available data from Trott and Bergen (2023), in which participants performed a primed sensibility judgment task. Specifically, they were asked to judge whether a target sentence was a sensible sentence of English (e.g., “She liked the marinated lamb”) after reading a prime sentence containing the same ambiguous word. The prime sentence conveyed either the same meaning (e.g., “roasted lamb”) or a different meaning (e.g., “friendly lamb”). Here, we aggregated across individual trials to calculate the mean accuracy and mean Log RT (for correct responses only) for each item.

#### Does sensorimotor distance account for online processing?

We first asked whether sensorimotor distance exhibited a significant relationship with either mean Log RT or accuracy. In each case, we constructed a linear mixed effects model with the human behavioral measure as a dependent variable, a fixed effect of sensorimotor distance, and random intercepts for each word. We found that sensorimotor distance was a significant predictor for both accuracy [β=-0.46, SE=0.08, p<.001] and mean Log RT [β=0.23, SE=0.04, p<.001]. That is, pairs of contexts with more distinct sensorimotor profiles were associated with lower accuracies and slower response times. Together, these results suggest that sensorimotor distance captures variance in online processing of ambiguous words.

#### Evaluating sensorimotor vs. distributional distance

We then asked which continuous measure of contextual distance best accounted for variance in response time and accuracy. As in Section “Sensorimotor distance and graded relatedness”, we compared the predictive power of sensorimotor distance, distributional distance, and a hybrid model containing both measures. For each layer of each LM, we fit a regression model predicting either accuracy or mean Log RT from distributional distance alone, and computed AIC; we also computed the AIC for a hybrid model containing both measures (along with the AIC for a model containing sensorimotor distance alone). We again calculated $\Delta AIC_{SM}$ ($AIC_{D} - AIC_{SM}$, i.e., asking which distance measure alone provided a better fit) and $\Delta AIC_{hybrid}$ ($AIC_{D} - AIC_{hybrid}$, i.e., asking whether the combination of both distance measures provided a better fit than distributional distance alone). In each case, a positive ΔAIC reflects worse performance by distributional distance alone. We report results for the best-performing layer of each LM.

Considering just $\Delta AIC_{SM}$, we found support for distributional distance over sensorimotor distance for 9 of the 12 LMs tested for accuracy, and 11 for Log RT. That is, for both dependent measures, distributional distance provided a better fit for a majority of LMs. At the same time, we also found that the *hybrid* models represented an improvement over distributional distance alone for 7 LMs when predicting accuracy, and 6 LMs when predicting Log RT. A comparison of the relative predictive power of each statistical model is illustrated in Fig. 8. Together, these results suggest that distributional measures of contextual similarity are better predictors of online processing in a primed sensibility judgment task than are sensorimotor measures – yet they also point to the predictive value of *both* sources of information.

#### Continuous measures and discrete sense boundaries

A key result from Trott and Bergen (2023) was the finding that continuous measures of contextual similarity did not fully account for the categorical effect of sense boundaries. This was interpreted as consistent with the hypothesis that human semantic representations exhibit categorical structure (i.e., discrete “senses”) on top of an underlying continuous meaning-space. A key limitation of that result, however, was that it relied primarily on a distributional measure of continuous distance; one possibility, therefore, is that the variance formerly attributed to categorical factors (i.e., sense boundaries) could instead be attributable to other continuous factors not captured by distributional statistics – such as sensorimotor similarity.

Here, we extended the primary analysis from Trott and Bergen (2023). Building on the results from Section “Evaluating sensorimotor vs. distributional distance” above, we compared the *AIC* of the *hybrid* models (i.e., containing both distributional distance and sensorimotor distance) to the *AIC* of a statistical model containing only sense boundaries, as well as a statistical model containing all three predictors. For all LMs tested – and both dependent measures – we found that the hybrid model containing both distributional distance and sensorimotor distance did *not* fully account for the effect of sense boundaries. That is, even accounting for two different operationalizations of continuous contextual similarity failed to explain away the previously observed effect of sense boundaries. These results are therefore consistent with past work suggesting that human semantic representations are best predicted by a combination of continuous and categorical factors (Trott & Bergen, 2023).

## Case study: Can large language models help scale the CS norms?

These promising use cases for the CS norms lead naturally to the issue of how to extend them to increase their scope and scale (see Section “Expanding the norms”). Here, we test the effectiveness of prompting LLMs to generate these norms. We use the term “LLM” to distinguish such models from the relatively smaller LMs tested in Section “Comparing distributional and sensorimotor similarity”: LLMs are typically identified by their size (i.e., billions or trillions of parameters) and large quantities of training data (i.e., hundreds of billions or even trillions of words) – and, in recent years, specialized training regimes (e.g., Reinforcement Learning with Human Feedback).

A growing body of work has investigated whether such LLMs can be used to help *augment* datasets of psycholinguistic norms automatically (Brysbaert, Martínez, & Reviriego, 2024; Chersoni et al., 2020; Conde et al., 2025; Hagihara & Miyazawa, 2026; Kewenig et al., 2025; Martínez et al., 2024; Martínez, Conde, Reviriego, & Brysbaert, 2025b; Trott, 2024a, b; Turton et al., 2020; Utsumi, 2020; Xu et al., 2025). A large subset of this work focuses on norms relating to sensorimotor strength, such as concreteness (Kewenig et al., 2025; Martínez et al., 2024; Martínez, Conde, Reviriego, & Brysbaert, 2025a), body interactiveness (Hagihara & Miyazawa, 2026), and even specific sensorimotor dimensions (Xu et al., 2025; Trott, 2024a). In the sections below, we provide a brief explanation of existing work on this topic (Section “Background and existing work”), including an analysis of the CS norms specifically (Trott, 2024a); then, using existing data, we conduct several exploratory analyses asking whether LLM-generated norms could in principle be substituted for the human norms (Section “Substitution analyses”). As noted, these analyses are exploratory and are not intended to be exhaustive; they should therefore be treated with caution. However, they do provide an initial picture of whether and to what extent LLMs could facilitate scaling of contextualized norms.

### Background and existing work

As noted above, several studies have investigated the ability of LLMs to produce norms about sensorimotor associations specifically (Hagihara & Miyazawa, 2026; Kewenig et al., 2025; Trott, 2024a; Wu, Conde, Reviriego, & Brysbaert, 2026; Xu et al., 2025). Typically, norms are elicited from LLMs by adapting the instructions given to human participants and prompting a given LLM (e.g., GPT-4) to produce a number in the desired range (e.g., between 0 and 5) (Conde et al., 2025). The resulting “norms” can then be compared to human norms, either directly (i.e., in terms of their correlation strength or systematic patterns of errors) or indirectly (i.e., in terms of whether they can *substitute* for human norms in a statistical analysis without meaningful changes in the fit parameters).

Although LLMs have enjoyed success producing a variety of psycholinguistic norms (Conde et al., 2025), past work suggests that LLMs may struggle to produce judgments of sensorimotor strength for words in isolation (Xu et al., 2025). This is consistent with the hypothesis that these models’ lack of sensorimotor grounding places limits on their ability to represent the meanings of more concrete words. However, a recent study (Wu et al., 2026) suggests that *fine-tuning* models on a subset of the LS norms can substantively improve their performance, perhaps driving a reorganization of their semantic space.

Most relevant to the current work, Trott (2024a) assessed the ability of GPT-4 to reproduce the human CS norms specifically. LLM-generated norms correlated moderately well with human judgments (ranging from ρ=0.45 to ρ=0.75). Correlations were stronger for perception than action dimensions, which could be explained by one of two factors: first, it is possible that information about perceptual strength is easier to glean from the distributional statistics of language, or from context; alternatively, *agreement* may simply be higher for perception than action dimensions overall, as we report in Section “Inter-annotator agreement”. Notably, in some cases, LLM-generated judgments aligned better with the human mean than did individual human judgments, consistent with other work suggesting that LLMs capture the “wisdom of the crowd” (Trott, 2024b). Finally, Trott (2024a) report that an LLM-derived measure of *sensorimotor distance* was predictive of human relatedness judgments, suggesting that the LLM-generated norms (like the human norms) exhibit some degree of predictive utility.

**Fig. 9:**
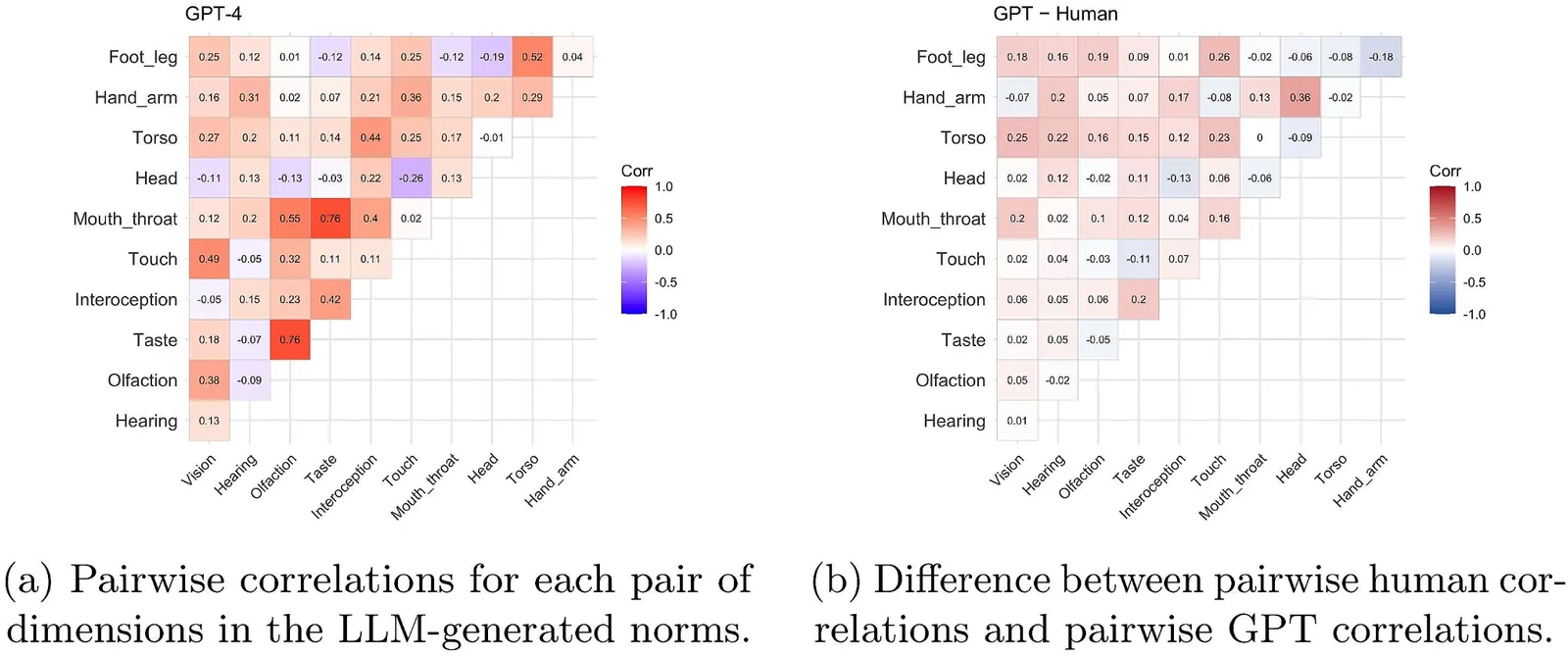
Patterns of between-dimension correlations for the LLM-generated norms (*left*) and their comparison to the human-generated norms (*right*). See Fig. 1b for the correlations using the original human ratings

Below, we expand on the analyses from Trott (2024a) in two ways. First, beyond investigating whether LLM-generated norms correlate with human-generated norms, we ask whether both datasets show qualitatively similar patterns of *between-dimension* correlation (Section “Between-dimension comparison”). We then conduct several *substitution analyses*, in which LLM-generated norms are substituted for human norms in a statistical analysis to determine whether any theoretical or practical inferences would meaningfully change (Section “Substitution analyses”). Specifically, we ask whether LLM-generated norms show similar patterns of contextual variance as a function of ambiguity type (Section “Do different senses have more distinct sensorimotor profiles?”) and whether they reproduce the pattern of meaning dominance, i.e., in which more dominant meanings exhibit higher sensorimotor strength overall (Section “Dominance and sensorimotor strength”).

### Between-dimension comparison

As noted above, Trott (2024a) elicited contextualized sensorimotor judgments from GPT-4, a closed-source LLM. Briefly, GPT-4 was prompted with instructions adapted as closely as possible from those given to human participants in the original CS norms collection (Section “Contextualized sensorimotor norms”), and was asked to rate the salience of each sensorimotor dimension on the same 0–5 scale; each sentence × dimension cell was elicited as an independent trial with temperature set to 0. See the original paper for the full details of the elicitation procedure.

Here, we asked whether the LLM-generated CS norms exhibited the same patterns of *between-dimension* correlation identified in the human norms (Fig. 1b). As in Section “Characterizing the contextualized sensorimotor norms”, we calculated Pearson’s correlation between each pair of dimensions. We observed particularly strong correlations between olfaction and taste (r=0.81), as well as between mouth/throat and taste (r=0.64) and foot/leg and torso (r=0.61); see Fig. 9a for the full set of pairwise comparisons. We quantified the degree of alignment between the pairwise correlations for the LLM-generated norms and human-generated norms using a Mantel test, comparing the observed correlations between the two matrices to a null distribution generated by 9999 random permutations. The two matrices were significantly aligned whether measured using Pearson’s correlation (Mantel r=0.88, p<0.001) or Spearman’s correlation (Mantel ρ=0.81, p<0.001). Figure 9b depicts the differences for each pair of dimensions. The largest absolute differences were observed for the following pairwise correlations: head and hand/arm (Δr=0.36) and touch and foot/leg (Δr=0.26). In both cases, the correlation was stronger for the LLM-generated norms than for the human-generated norms.

### Substitution analyses

We then asked whether LLM-generated norms could *substitute* for human norms in a statistical analysis without key theoretical or practical inferences changing. Previous work had already found that an LLM-generated measure of sensorimotor distance was predictive of human relatedness judgments (Trott, 2024a), so we focused here on two novel results.

**Fig. 10:**
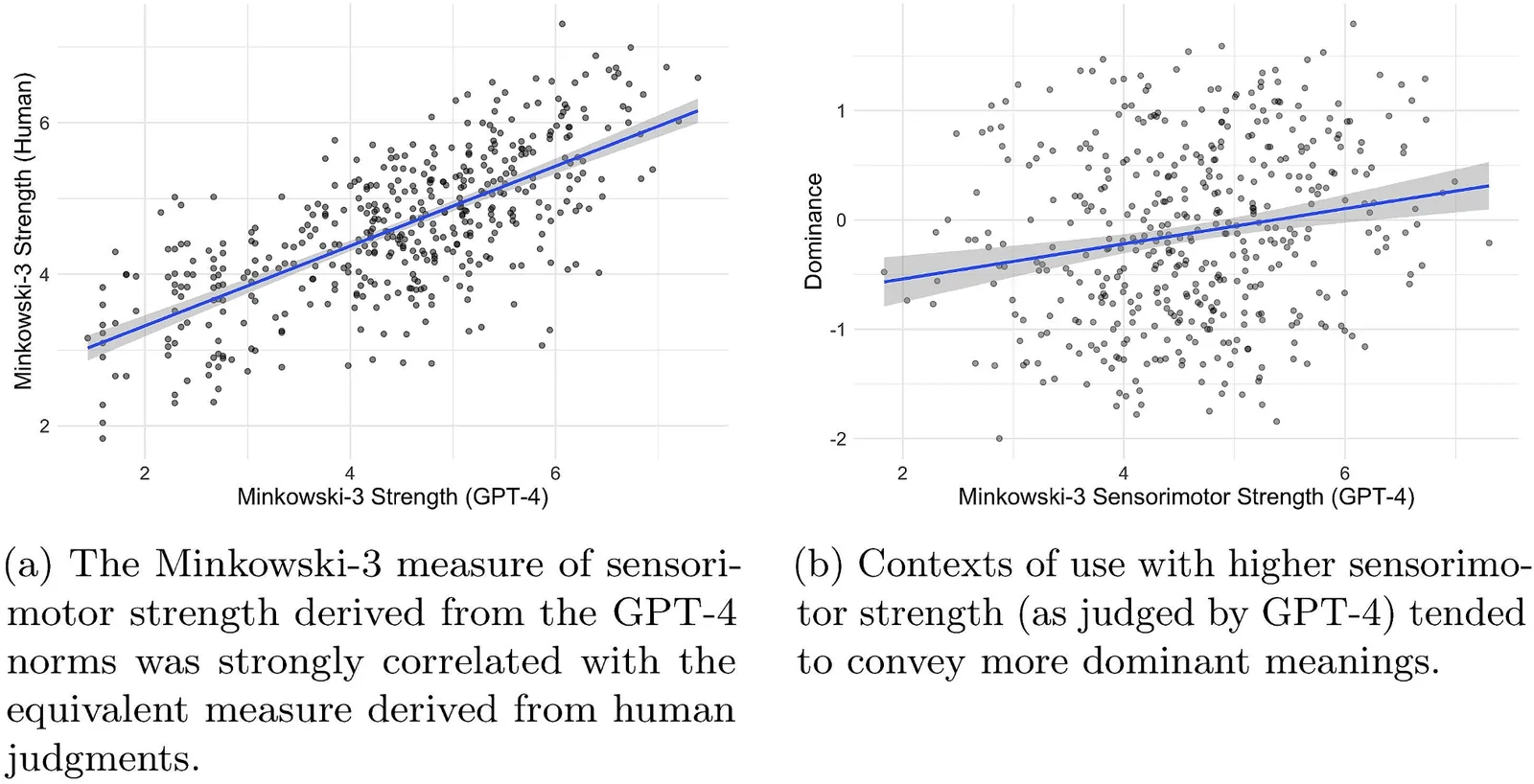
Contextualized sensorimotor norms elicited from GPT-4 resulted in aggregate measures of *sensorimotor strength* that correlated strongly with the equivalent measure from humans (*left*), and which were also predictive of meaning dominance (*right*), albeit less so than human-derived measures of sensorimotor strength

#### Sensorimotor distance and word senses

First, we aimed to replicate the analyses in Section “Do different senses have more distinct sensorimotor profiles?”: we asked whether LLM-derived *sensorimotor distance* (calculated using the same procedure as human-derived sensorimotor distance) reliably co-varied in the expected direction with the presence of a sense boundary, and whether these differences were larger for homonyms than polysemes. We constructed a linear mixed effects model with LLM-derived sensorimotor distance as a dependent variable, a fixed effect of sense boundary, by-word random slopes for the effect of sense boundary, and random intercepts for each word. This full model explained significantly more variance than a reduced model omitting only the effect of sense boundary $[\chi ^2(1) = 66.91, p < 0.001]$. As expected, same sense contexts were associated with a lower sensorimotor distance than different sense contexts [β=-0.08,SE=0.01,p<0.001].

We then asked whether this effect was larger for homonyms than polysemes. We fit a linear mixed effects model with LLM-derived sensorimotor distance as a dependent variable, fixed effects of sense boundary and ambiguity type (homonymy vs. polysemy), an interaction between those factors, by-word random slopes for the effect of sense boundary, and random intercepts for each word. This model explained significantly more variance than a reduced model omitting only the interaction $[\chi ^2(1) = 10.15, p = 0.001]$. The reference level (the intercept) corresponded to different sense homonym pairs, for which the model estimated a sensorimotor distance of 0.16. Same sense homonyms exhibited significantly lower LLM-derived sensorimotor distance [β=-0.12,SE=0.01,p<0.001], as did different sense polysemes [β=-0.05,SE=0.02,p=0.01]. Crucially, the interaction term was significant and positive [β=0.06,SE=0.02,p=0.001], indicating (as with the human-derived norms) that the gap between homonyms and polysemes was significantly smaller for same sense pairs than for different sense pairs.

Together, these results suggest that the key inferences drawn in Section “Do different senses have more distinct sensorimotor profiles?” – namely, that different-sense contexts have more distinct sensorimotor profiles than same-sense contexts, and that this gap is larger for homonyms than polysemes – hold when LLM-derived norms are substituted for human-derived norms.

#### Sensorimotor strength and meaning dominance

We then aimed to replicate the analyses in Section “Dominance and sensorimotor strength”, i.e., the finding that meanings with higher sensorimotor strength overall were also more *dominant*. As in Section “Dominance and sensorimotor strength”, we operationalized sensorimotor strength in two ways using the LLM-generated norms: first, using the maximum strength across all dimensions for a given context of use; and second, using Minkowski-3 strength (the cube root of the sum of cubed strength values across all 11 dimensions). Both measures were relatively aligned with equivalent measures derived from the human norms: max strength (r=0.65,p<0.001) and Minkowski-3 strength (r=0.7,p<0.001); see also Fig. 10a.

As in Section “Dominance and sensorimotor strength”, for each metric, we built a linear mixed effects model with dominance as a dependent variable, fixed effects of contextualized sensorimotor strength, random intercepts for each word, and two covariates reflecting the decontextualized sensorimotor strength for that word (i.e., from the LS norms). We found that the LLM-derived measure significantly improved model fit for Minkowski-3 strength $[\chi ^2(1) = 5.88, p = 0.02]$, but not for the max strength metric (p=0.2). As in the human analysis, higher Minkowski-3 strength was associated with more dominant meanings [β=0.08,SE=0.03,p=0.02]. Notably, however, the magnitude of this relationship was smaller than for the human norms (β=0.28). This suggests that while LLM-generated norms may be able to reproduce the qualitative direction of results (at least with the Minkowski-3 measure), they may underestimate the strength of the relationship between sensorimotor strength and dominance.

### Discussion

Taken together, the analyses above suggest that LLM-derived sensorimotor could in principle serve as a useful (though imperfect) complement to human-derived norms. GPT-4 recovered the broad organizational structure of the CS norms, i.e., capturing which dimensions correlated with each other most strongly (Section “Between-dimension comparison”). Moreover, when LLM-derived measures were substituted for human-derived measures, we replicated the effect of sense boundaries and ambiguity type on sensorimotor distance: namely, contexts corresponding to distinct senses have more distinct sensorimotor profiles, and this effect is larger for homonyms than polysemes. At the same time, the replication of the relationship between contextualized sensorimotor strength and meaning dominance had mixed results: we replicated the effect with only one of the measures of sensorimotor strength (Minkowski-3), and the magnitude of this relationship was smaller than when using a human-derived measure. These issues are discussed in more detail in Section “Expanding the norms”.

## General discussion

Theories of semantic representation differ in the emphasis they place on sensorimotor experience (Bergen, 2012; Pulvermüller, 1999; Barsalou, 1999) versus distributional information (Lenci, 2018). One piece of evidence for embodied theories comes from empirical analyses demonstrating the utility of sensorimotor *norms* in predicting human behavior (Lynott et al., 2019; Vinaya et al., 2025). Yet one limitation of most existing norming databases is that they were collected for words in isolation – even though most words mean different things in different contexts (Rodd et al., 2004). We addressed this limitation by creating the contextualized sensorimotor (CS norms), a dataset of sensorimotor judgments about 112 ambiguous English words in four minimal pair sentence contexts.

We first demonstrated that these ratings reliably capture sensorimotor variation across contexts relative to the original Lancaster sensorimotor norms (Fig. 3). For instance, “fish market” evokes smell and taste, while “stock market” does not (Fig. 2). Moreover, homonyms displayed more variance in their sensorimotor associations across contexts than polysemes (Sections “Within-word sensorimotor variance” and “Sensorimotor distance and word senses”). Together, these findings provide evidence that sensorimotor associations do, in fact, vary across a word’s context of use – and that this variance is to a large extent systematic and predictable (e.g., different sense contexts have more distinct sensorimotor associations than same sense contexts).

We then used these norms to address practical and theoretical questions about the representation of ambiguous words. First, in Section “Sense dominance and deviation from the Lancaster norms”, we found that sensorimotor norms for ambiguous words *in isolation* likely reflect the “dominant” or more psychologically salient meaning of those words. That is, the decontextualized Lancaster sensorimotor norms were more similar to the contextualized ratings of more dominant meanings in the CS norms than ratings for subordinate meanings; dominant meanings also tended to have higher sensorimotor strength overall, echoing past work (Gilhooly & Logie, 1980). From a theoretical perspective, these results point to a potentially important role for sensorimotor grounding in shaping the psychological dominance of a particular meaning – either directly or indirectly (e.g., if more concrete meanings are also learned earlier). Practically, they also suggest that ratings for words in isolation may be driven by the features associated with the dominant interpretation of that word. For some purposes, this might be desirable (i.e., if researchers are primarily interested in the features that first “come to mind” when encountering a lexical item in isolation). In other cases, however, researchers might want to know about the features associated with particular *meanings* of an ambiguous wordform. If the wordform is not presented in a disambiguating context, it is difficult to disentangle whether the resulting norms reflect only the dominant meaning or a mixture of meanings – as might be the case if different speakers with different backgrounds or expertise interpret the target word differently (Wiley, George, & Rayner, 2018).

The finding that homonyms have more distinct sensorimotor profiles than polysemes (on average) is also of theoretical interest, providing an account of what might make homonyms more distinct in their meanings than polysemes. Of course, the broad category of polysemy can also be decomposed into different types, such as regular vs. irregular polysemy (Pustejovsky, 1991; Rabagliati, Marcus, & Pylkkänen, 2011; Srinivasan & Rabagliati, 2015), which in turn may have distinct implications about how children and adults learn and represent the meanings of words (Floyd & Goldberg, 2021; Klepousniotou & Baum, 2007; Klepousniotou, Titone, & Romero, 2008; Rabagliati, Marcus, & Pylkkänen, 2010; Srinivasan, Berner, & Rabagliati, 2019). The CS norms were not explicitly designed to control for these types, but there are clear examples of polysemy involving systematic, regular relations (e.g., “marinated lamb” vs. “friendly lamb”), perceptual similarity (e.g., “traffic cone” vs. “waffle cone”), and metaphor (e.g., “drain the account” vs. “drain the pool”). Future work could explore whether distinct types of polysemous relations can be identified on the basis of their contextualized sensorimotor variance – for instance, whether metaphoric extensions systematically reduce sensorimotor strength relative to their sources, or whether regular polysemy preserves more sensorimotor structure than irregular polysemy.

We also asked whether the CS norms predicted the *relationship* between two contexts of use, including whether two contexts conveyed the same sense (Section “Sensorimotor distance and word senses”), the relatedness between those contexts, as judged by human raters (Section “Sensorimotor distance and graded relatedness”), and human behavioral responses to a target context following a prime context (Section “Sensorimotor distance and ambiguity processing”). We compared the predictive power of *sensorimotor distance* (i.e., the difference in sensorimotor profile across usage contexts) to *distributional distance*, as operationalized by the embeddings from transformer language models ranging in size and capability. Consistent with past work (Andrews et al., 2009, 2014; Davis & Yee, 2021; Kiros et al., 2018; Louwerse, 2018; Vinaya et al., 2025), we found evidence supporting a *hybrid* theory of semantic representations. That is, the relationship between usage contexts was best predicted by a *combination* of sensorimotor and distributional metrics, suggesting that lexical representations integrate both sources of information. We also replicated and extended past work suggesting that human semantic representations are further shaped by categorical factors (Trott & Bergen, 2023): even accounting for both distributional and sensorimotor distance measures, the presence or absence of a sense boundary between contexts explained additional variance in human behavior. Future work could investigate whether these paths to meaning play a differential role in different contexts or different types of words, e.g., whether sensorimotor experience plays a relatively stronger role in shaping our representations of concrete vs. abstract words (Lupyan & Winter, 2018; Borghi et al., 2019).

Finally, we extended past work (Trott, 2024a) asking whether large language models (LLMs) could augment datasets of psycholinguistic norms (Section “Case study: Can large language models help scale the CS norms?”). Using an existing dataset of norms produced by GPT-4 (Trott, 2024a), we found that LLM-generated norms exhibited similar patterns of correlations between sensorimotor dimensions, as well as similar patterns of contextual variance: contexts conveying different senses had more divergent sensorimotor profiles than same-sense contexts, with the largest divergences for homonyms. We also partially replicated the relationship between sensorimotor strength and meaning dominance, though the effect was smaller and limited to only one of the metrics. These results provide an initial demonstration that LLM-derived norms can support *some* of the theoretical inferences drawn from the CS norms, while also suggesting potential limitations. Crucially, none of these analyses would have been possible without a carefully controlled human-derived dataset against which to evaluate the LLM-generated norms.

### Expanding the norms

A primary limitation of the current work is *scope*. The CS norms are relatively small: 448 sentences (112 words, with four sentences each), in English only. The words themselves may not be representative of ambiguous words more generally, and the sentences were designed for the purpose of experimental control rather than naturalism. In contrast, the Lancaster sensorimotor norms contain judgments of almost 40,000 English words (Lynott et al., 2019), as well as French (Miceli et al., 2021), Dutch (Speed & Brybaert, 2021), and more. Moreover, other norming datasets contain an even richer distribution of interpretable features (Binder et al., 2016), and there is growing acknowledgment of the importance of other aspects (e.g., social) of linguistic grounding (Johns, 2021).

Having demonstrated the practical and theoretical utility of the CS norms on a small subset of English words, one obvious direction for future research would be to expand this dataset – including more words, more senses and sentences per word, a wider variety of sentences (i.e., both experimentally controlled and naturalistic sentences), additional languages, and additional features. Of course, scaling is costly and challenging, not least because there are, presumably, an *infinite* number of contexts in which any ambiguous word could occur. In contrast, ratings of individual words – while limited in the ways discussed earlier – provide a more finite target bounded by the size of the lexicon. Such ratings can also be more easily repurposed, since they are (by definition) not bound to specific contexts. One question, then, is whether and to what extent ratings about words in context can be *generalized*, such that a tractable number of contextualized ratings can support research about a space of larger possible usages.

#### Sense-level annotation

A useful starting point would be to augment existing lexical ambiguity datasets (Erk, McCarthy, & Gaylord, 2013; Haber & Poesio, 2021; Karidi, Zhou, Schneider, Abend, & Srikumar, 2021; Schlechtweg, Tahmasebi, Hengchen, Dubossarsky, & McGillivray, 2021) with sensorimotor judgments about each word’s context of use, allowing researchers to replicate the present analyses comparing the predictive power of sensorimotor and distributional distance. Such analyses could also inform questions about the role of sensorimotor knowledge in different kinds of polysemous relations, such as metonymy or metaphor (Winter & Srinivasan, 2022). One key benefit of annotating *sense-tagged* datasets in particular is that generalization could be achieved at the level of individual word senses, as opposed to specific linguistic contexts (which are more granular, but less generalizable) or wordforms (which, as argued throughout the paper, collapse across distinct meanings). Beyond the practical advantages, sense-level annotation of sensorimotor features could be used to identify psychologically meaningful sources of semantic variance across word meanings, bearing on debates about the discrete vs. continuous structure of semantic representations (Trott & Bergen, 2023).

#### Augmenting with large language models

As discussed in Section “Case study: Can large language models help scale the CS norms?”, another approach towards scaling the CS norms could lie in the use of large language models (LLMs). The case study reported in Section “Case study: Can large language models help scale the CS norms?” suggests that LLM-generated norms can in principle serve as a useful (though imperfect) complement to human-derived norms. It is worth noting that the analyses in Section “Case study: Can large language models help scale the CS norms?” used norms from GPT-4; while GPT-4 performed well on the norming task (Trott, 2024a), it is plausible that more recent models could perform better, given that LLMs have seen improvements along a number of theoretical and applied benchmarks in the last several years. Thus, the performance reported in Section “Case study: Can large language models help scale the CS norms?” could reasonably be seen as a “floor” on LLM norming performance, at least for datasets like the CS norms.

Here, we briefly discuss several methodological considerations for future work that aims to integrate LLMs into the norming process. Specifically, what steps can researchers take to improve or ensure the validity of LM-derived sensorimotor norms? And how (if at all) should researchers seek to integrate these norms into the scientific pipeline? Conde et al. (2025) provides an overview of relevant decision criteria for LM-based norming in general, including which LM to select and whether to *fine-tune* the LM on a subset of the target norms (Wu et al., 2026)

Of particular relevance to the current work is that Conde et al. (2025) emphasize validating LM-derived norms against a human “gold standard”: even small datasets (like the CS norms) can serve as a useful gold standard against which to evaluate LMs, providing initial evidence as to whether an LM’s judgments exhibit alignment with human judgments. Beyond correlation analyses, researchers can make use of *substitution analyses* (Section “Substitution analyses”), in which LM-derived norms are substituted for human norms in the context of a statistical analysis to determine whether any of the inferences would meaningfully change. If LM-derived norms lead to qualitatively (or even quantitatively) similar conclusions as human norms, researchers might feel more confident using them in the context of a lexicon-wide analysis or in norming experimental stimuli.

A complementary strategy involves *centaur* approaches that combine human and LM judgments, providing a stronger guarantee of validity (i.e., because of the human sample) while also enabling larger scale (i.e., because LMs are both cheaper and faster to use in this context). For example, Trott (2024b) finds that averaging LM-derived ratings with a small sample of human ratings can produce more reliable estimates than either source alone, especially where human inter-annotator agreement is low. More broadly, this approach reframes the question from whether LM-derived norms should “replace” human norms to the question of how best to integrate the two sources of information. Even if LMs are used in the process, datasets of human ratings – like the CS norms – remain crucial for validation and calibration.

## Figures and Tables

**Fig. 11:**
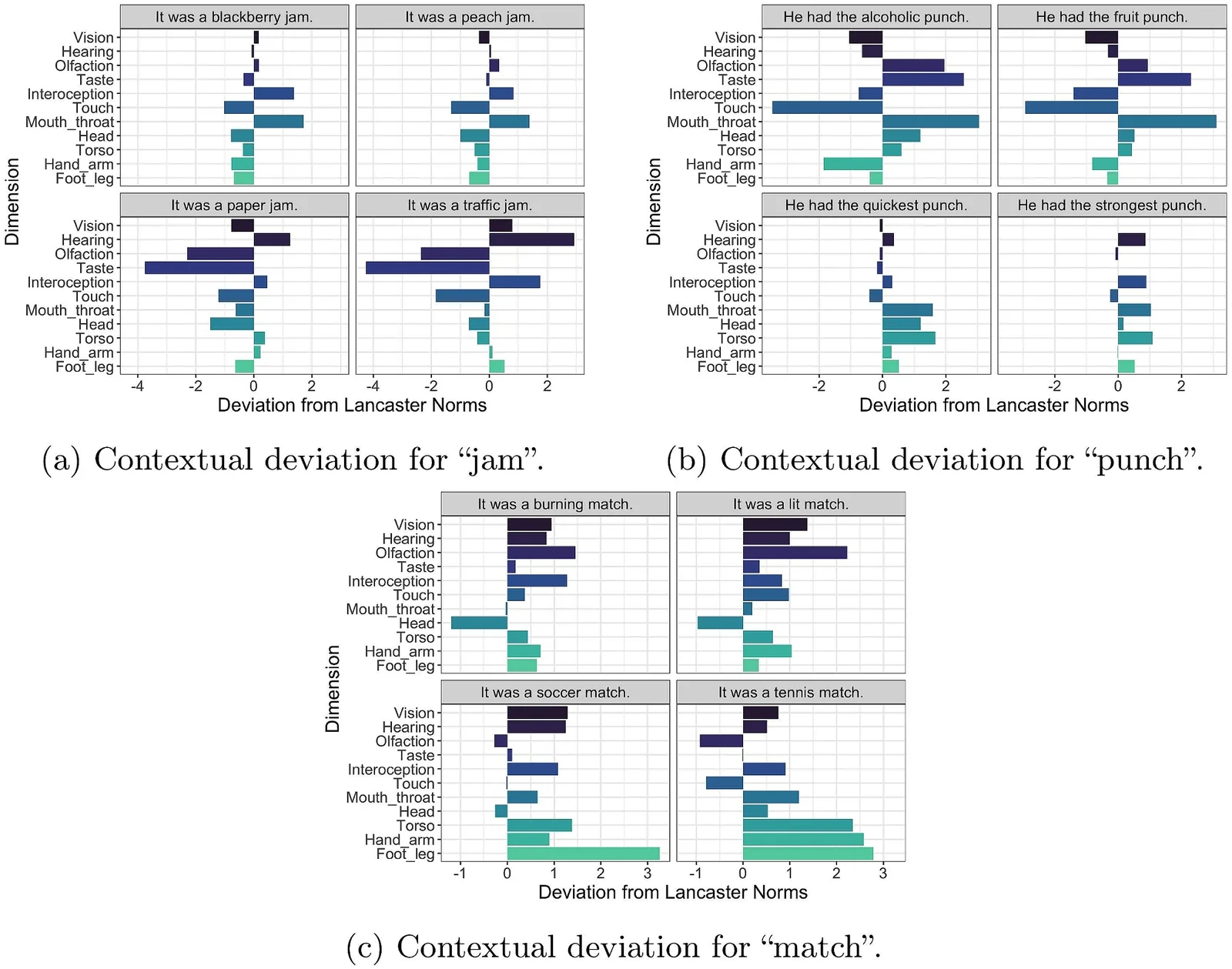
Deviation between the contextualized sensorimotor norms and the Lancaster sensorimotor norms for “jam”, “punch”, and “match”

## Data Availability

The contextualized sensorimotor norms are publicly available on GitHub (https://github.com/seantrott/cs_norms) and HuggingFace (https://huggingface.co/datasets/seantrott/cs_norms).

## Appendix A: Full list of language models

**Table 2:** Language models by family

Model	# Params
BERT-base-cased	∼ 108M
BERT-base-uncased	∼ 109M
BERT-base-multilingual-cased	∼ 178M
DistilBERT -base-uncased	∼ 66M
ALBERT-base-v1	∼ 12M
ALBERT-base-v2	∼ 12M
ALBERT-large-v2	∼ 18M
ALBERT-xlarge-v2	∼ 59M
ALBERT-xxlarge-v2	∼ 223M
RoBERTa-base	∼ 125M
RoBERTa-large	∼ 355M
XLM-RoBERTa-base	∼ 278M

## Appendix C: AIC for individual models

**Table 3:** AIC values for predicting sense boundaries using distributional distance alone or both distributional distance and sensorimotor distance (AIC for sensorimotor distance was 707)

Model	Distributional	Hybrid
ALBERT-base-v1	737	627
ALBERT-base-v2	626	564
ALBERT-large-v2	669	592
ALBERT-xlarge-v2	542	506
ALBERT-xxlarge-v2	457	429
BERT-base-cased	591	538
BERT-base-uncased	531	494
DistilBERT	604	554
Multilingual BERT	742	632
RoBERTa-base	569	514
RoBERTa-large	452	435
XLM-RoBERTa	810	689

**Table 4:** AIC values for predicting relatedness judgments using distributional distance alone or both distributional distance and sensorimotor distance (AIC for sensorimotor distance was 2134.78)

Model	Distributional	Hybrid
ALBERT-base-v1	2246	2053
ALBERT-base-v2	2139	1997
ALBERT-large-v2	2142	2002
ALBERT-xlarge-v2	1995	1878
ALBERT-xxlarge-v2	1901	1790
BERT-base-cased	2038	1911
BERT-base-uncased	1960	1855
DistilBERT	2056	1937
Multilingual BERT	2225	2062
RoBERTa-base	2018	1899
RoBERTa-large	1844	1759
XLM-RoBERTa	2277	2088

## Footnotes

1 The norms can be found on GitHub (https://github.com/seantrott/cs_norms) as well as HuggingFace (https://huggingface.co/datasets/seantrott/cs_norms).

2 Lynott et al. (2019) reported that m=3 was the optimal exponent for Minkowski metric in terms of predicting downstream behavioral measures.

## Code Availability

Analysis code can be found on GitHub: https://github.com/seantrott/cs_norms.

## Declarations

### Open Practices Statement

All code and data can be found in a publicly available repository (see above).

## References

[CR1] Andrews, M., Vigliocco, G., & Vinson, D. (2009). Integrating experiential and distributional data to learn semantic representations. *Psychological review,**116*(3), 463. 19618982 10.1037/a0016261

[CR2] Andrews, M., Frank, S., & Vigliocco, G. (2014). Reconciling embodied and distributional accounts of meaning in language. *Topics in cognitive science,**6*(3), 359–370. 24935903 10.1111/tops.12096

[CR3] Barsalou, L. W. (1999). Perceptual symbol systems. *Behavioral and Brain Sciences,* *22*(4), 577–660. 10.1017/S0140525X99002149 Retrieved 2020-11-24 https://www.cambridge.org/core/journals/behavioral-and-brain-sciences/article/perceptual-symbol-systems/C2D720D63C1CE3D7153F6BA473F9DD87https://www.cambridge.org/core/journals/behavioral-and-brain-sciences/article/perceptual-symbol-systems/C2D720D63C1CE3D7153F6BA473F9DD87. (Publisher: Cambridge University Press) 11301525

[CR4] Barsalou, L.W. (2008). Grounded Cognition. *Annual Review of Psychology*, *59*(1), 617–645, 10.1146/annurev.psych.59.103006.093639 Retrieved 2020-11-24 17705682

[CR5] Bates, D., Mächler, M., Bolker, B., Walker, S. (2015). Fitting linear mixed-effects models using lme4. *Journal of Statistical Software*, *67*(1), 1–48, 10.18637/jss.v067.i01

[CR6] Bender, E.M., & Koller, A. (2020). Climbing towards NLU: On meaning, form, and understanding in the age of data. In *Proceedings of the 58th annual meeting of the association for computational linguistics* (pp. 5185–5198). Online: Association for Computational Linguistics. Retrieved from https://aclanthology.org/2020.acl-main.463

[CR7] Bergen, B. (2012). *Louder Than Words: The New Science of How the Mind Makes Meaning*. New York, NY, USA: Basic Books. Retrieved 2020-11-24 http://ebookcentral.proquest.com/lib/ucsd/detail.action?docID=1028039

[CR8] Bergen, B., & Feldman, J. (2008). 16 - Embodied Concept Learning. P. Calvo, & A. Gomila (Eds.), *Handbook of Cognitive Science* (pp. 313–331). San Diego: Elsevier. Retrieved 2020-11-24 http://www.sciencedirect.com/science/article/pii/B9780080466163000165

[CR9] Binder, K., & Rayner, K. (1998). Contextual strength does not modulate the subordinate bias effect: Evidence from eye fixations and self-paced reading. *Psychonomic Bulletin & Review*, *5*(2), 271–276, Retrieved from https://link.springer.com/article/10.3758/BF03212950

[CR10] Binder, J., Conant, L. L., Humphries, C. J., Fernandino, L., Simons, S. B., Aguilar, M., & Desai, R. H. (2016). Toward a brain-based componential semantic representation. *Cognitive neuropsychology,**33*(3–4), 130–174. 27310469 10.1080/02643294.2016.1147426

[CR11] Bisk, Y., Holtzman, A., Thomason, J., Andreas, J., Bengio, Y., Chai, J., . . . Turian, J. (2020). Experience grounds language. In *Proceedings of the 2020 conference on empirical methods in natural language processing (emnlp)* (pp. 8718–8735). Online: Association for Computational Linguistics. Retrieved from https://aclanthology.org/2020.emnlp-main.703

[CR12] Borghi, A. M., Barca, L., Binkofski, F., Castelfranchi, C., Pezzulo, G., & Tummolini, L. (2019). Words as social tools: Language, sociality and inner grounding in abstract concepts. *Physics of life reviews,**29*, 120–153. 30573377 10.1016/j.plrev.2018.12.001

[CR13] Brysbaert, M., Stevens, M., De Deyne, S., Voorspoels, W., & Storms, G. (2014). Norms of age of acquisition and concreteness for 30,000 dutch words. *Acta psychologica,**150*, 80–84. 24831463 10.1016/j.actpsy.2014.04.010

[CR14] Brysbaert, M., Warriner, A. B., & Kuperman, V. (2014). Concreteness ratings for 40 thousand generally known english word lemmas. *Behavior research methods,**46*(3), 904–911. 24142837 10.3758/s13428-013-0403-5

[CR15] Brysbaert, M., Martínez, G., & Reviriego, P. (2024). Moving beyond word frequency based on tally counting: Ai-generated familiarity estimates of words and phrases are an interesting additional index of language knowledge. *Behavior Research Methods,**57*(1), 28. 39733132 10.3758/s13428-024-02561-7

[CR16] Burnham, K. P., & Anderson, D. R. (2004). Multimodel inference: understanding aic and bic in model selection. *Sociological methods & research,**33*(2), 261–304.

[CR17] Campbell, E., Casillas, R., & Bergelson, E. (2024). The role of vision in the acquisition of words: Vocabulary development in blind toddlers. *Developmental Science,**27*(4), e13475. 38229227 10.1111/desc.13475

[CR18] Chang, T.A., Arnett, C., Tu, Z., & Bergen, B.K. (2024). When is multilinguality a curse? language modeling for 250 high- and low-resource languages. In Y. Al-Onaizan, M. Bansal, & Y.- N. Chen (Eds.), *Proceedings of the 2024 conference on empirical methods in natural language processing* (pp. 4074–4096). Miami, Florida, USA: Association for Computational Linguistics. Retrieved from https://aclanthology.org/2024.emnlp-main.236/

[CR19] Chersoni, E., Xiang, R., Lu, Q., & Huang, C.- R. (2020). Automatic learning of modality exclusivity norms with crosslingual word embeddings. In *Proceedings of the 9th joint conference on lexical and computational semantics* (pp. 32–38). Barcelona, Spain (Online): Association for Computational Linguistics. Retrieved from https://aclanthology.org/2020.starsem-1.4

[CR20] Coltheart, M. (1981). The mrc psycholinguistic database. *The Quarterly Journal of Experimental Psychology Section A,**33*(4), 497–505.

[CR21] Conde, J., Grandury, M., Fu, T., Arriaga, C., Martínez, G., Clark, T., & Brysbaert, M. (2025). Adding llms to the psycholinguistic norming toolbox: A practical guide to getting the most out of human ratings arXiv:2509.14405 arXiv preprint 10.3758/s13428-026-03129-3 PMC13407732 42509529

[CR22] Connell, L., & Lynott, D. (2012). Strength of perceptual experience predicts word processing performance better than concreteness or imageability. *Cognition,**125*(3), 452–465. 22935248 10.1016/j.cognition.2012.07.010

[CR23] Connell, L., & Lynott, D. (2016). Do we know what we’re simulating? information loss on transferring unconscious perceptual simulation to conscious imagery. *Journal of Experimental Psychology: Learning, Memory, and Cognition,**42*(8), 1218. 26866656 10.1037/xlm0000245

[CR24] Ćoso, B., Guasch, M., Ferré, P., & Hinojosa, J. A. (2019). Affective and concreteness norms for 3,022 croatian words. *Quarterly Journal of Experimental Psychology,**72*(9), 2302–2312. 10.1177/1747021819834226 30744508

[CR25] Davis, C. P., & Yee, E. (2021). Building semantic memory from embodied and distributional language experience. *Wiley Interdisciplinary Reviews: Cognitive Science,**12*(5), e1555. 33533205 10.1002/wcs.1555

[CR26] De Leeuw, J. R. (2015). jspsych: A javascript library for creating behavioral experiments in a web browser. *Behavior research methods,**47*(1), 1–12. 24683129 10.3758/s13428-014-0458-y

[CR27] Devlin, J., Chang, M., Lee, K., & Toutanova, K. (2018). BERT: pre-training of deep bidirectional transformers for language understanding. CoRR, Retrieved from arXiv:1810.04805

[CR28] Duffy, S. A., Morris, R. K., & Rayner, K. (1988). Lexical ambiguity and fixation times in reading. *Journal of memory and language,**27*(4), 429–446.

[CR29] Elman, J.L. (2009). On the meaning of words and dinosaur bones: Lexical knowledge without a lexicon. *Cognitive science*, *33*(4), 547–582. Retrieved from https://onlinelibrary.wiley.com/doi/full/10.1111/j.1551-6709.2009.01023.x 10.1111/j.1551-6709.2009.01023.x PMC2721468 19662108

[CR30] Erk, K., McCarthy, D., & Gaylord, N. (2013). Measuring word meaning in context. *Computational Linguistics,**39*(3), 511–554.

[CR31] Fernandino, L., Tong, J.- Q., Conant, L.L., Humphries, C.J., & Binder, J.R. (2022). Decoding the information structure underlying the neural representation of concepts. *Proceedings of the National Academy of Sciences,* *119*(6), e2108091119. 10.1073/pnas.2108091119 PMC8832989 35115397

[CR32] Floyd, S., & Goldberg, A. E. (2021). Children make use of relationships across meanings in word learning. *Journal of Experimental Psychology: Learning, Memory, and Cognition,**47*(1), 29. 32105145 10.1037/xlm0000821

[CR33] Fodor, J. (1975). *The language of thought*. Harvard University Press.

[CR34] Gilhooly, K. J., & Logie, R. H. (1980). Meaning-dependent ratings of imagery, age of acquisition, familiarity, and concreteness for 387 ambiguous words. *Behavior Research Methods & Instrumentation,**12*(4), 428–450.

[CR35] Glenberg, A. M., & Kaschak, M. P. (2002). Grounding language in action. *Psychonomic bulletin & review,**9*(3), 558–565. 12412897 10.3758/bf03196313

[CR36] Haber, J., & Poesio, M. (2020). Assessing polyseme sense similarity through co-predication acceptability and contextualised embedding distance. In *Proceedings of the 9th joint conference on lexical and computational semantics* (pp. 114–124). Barcelona, Spain (Online): Association for Computational Linguistics. Retrieved from https://aclanthology.org/2020.starsem-1.12

[CR37] Haber, J., & Poesio, M. (2021). Patterns of polysemy and homonymy in contextualised language models. In *Findings of the association for computational linguistics: Emnlp 2021* (pp. 2663–2676). Punta Cana, Dominican Republic: Association for Computational Linguistics. Retrieved from https://aclanthology.org/2021.findings-emnlp.226

[CR38] Hagihara, H., & Miyazawa, K. (2026). How well do large language models mirror human cognition of word concepts?: A comparison of psychological ratings for early-acquired english words. *Behavior Research Methods,**58*(2), 58. 41629738 10.3758/s13428-025-02938-2 PMC12864368

[CR39] Harnad, S. (1990). The symbol grounding problem. *Physica D: Nonlinear Phenomena,**42*(1–3), 335–346.

[CR40] Harris, Z. S. (1954). *Distributional structure. Word,**10*(2–3), 146–162.

[CR41] Jia, C., Yang, Y., Xia, Y., Chen, Y. T., Parekh, Z., Pham, H., & Duerig, T. (2021). Scaling up visual and vision-language representation learning with noisy text supervision. *International conference on machine learning* (pp. 4904–4916).

[CR42] Johns, B. T. (2021). Distributional social semantics: Inferring word meanings from communication patterns. *Cognitive Psychology,**131*, 101441. 34666227 10.1016/j.cogpsych.2021.101441

[CR43] Jones, C. R., & Bergen, B. (2024). Does word knowledge account for the effect of world knowledge on pronoun interpretation? *Language and Cognition,**16*(4), 1182–1213.

[CR44] Jones, C. R., Chang, T. A., Coulson, S., Michaelov, J. A., Trott, S., & Bergen, B. (2022). Distrubutional semantics still can’t account for affordances. In *Proceedings of the annual meeting of the cognitive science society* (Vol. 44).

[CR45] Karidi, T., Zhou, Y., Schneider, N., Abend, O., & Srikumar, V. (2021). Putting words in BERT’s mouth: Navigating contextualized vector spaces with pseudowords. In *Proceedings of the 2021 conference on empirical methods in natural language processing* (pp. 10300–10313). Online and Punta Cana, Dominican Republic: Association for Computational Linguistics. Retrieved from https://aclanthology.org/2021.emnlp-main.806

[CR46] Kennington, C. (2021). Enriching language models with visually-grounded word vectors and the Lancaster sensorimotor norms. In *Proceedings of the 25th conference on computational natural language learning* (pp. 148–157). Online: Association for Computational Linguistics. Retrieved from https://aclanthology.org/2021.conll-1.11

[CR47] Kewenig, V., Skipper, J. I., & Vigliocco, G. (2025). A multimodal transformer-based tool for automatic generation of concreteness ratings across languages. *Communications Psychology,**3*(1), 100. 40629107 10.1038/s44271-025-00280-z PMC12238627

[CR48] Kim, W., Son, B., Kim, I. (2021). Vilt: Vision-and-language transformer without convolution or region supervision. In *International conference on machine learning* (pp. 5583–5594).

[CR49] Kiros, J., Chan, W., & Hinton, G. (2018). Illustrative language understanding: Large-scale visual grounding with image search. In *Proceedings of the 56th annual meeting of the association for computational linguistics (volume 1: Long papers)* (pp. 922–933). Melbourne, Australia: Association for Computational Linguistics. Retrieved from https://aclanthology.org/P18-1085

[CR50] Klepousniotou, E., & Baum, S. R. (2007). Disambiguating the ambiguity advantage effect in word recognition: An advantage for polysemous but not homonymous words. *Journal of Neurolinguistics,**20*(1), 1–24.

[CR51] Klepousniotou, E., Titone, D., & Romero, C. (2008). Making sense of word senses: the comprehension of polysemy depends on sense overlap. *Journal of Experimental Psychology: Learning, Memory, and Cognition,**34*(6), 1534. 18980412 10.1037/a0013012

[CR52] Lan, Z., Chen, M., Goodman, S., Gimpel, K., Sharma, P., & Soricut, R. (2019). ALBERT: A lite BERT for self-supervised learning of language representations. CoRR, Retrieved from arXiv:1909.11942

[CR53] Lenci, A. (2018). Distributional models of word meaning. *Annual review of Linguistics,**4*(1), 151–171.

[CR54] Li, J., & Joanisse, M. F. (2021). Word senses as clusters of meaning modulations: A computational model of polysemy. *Cognitive Science,**45*(4), e12955. 33873247 10.1111/cogs.12955

[CR55] Liu, Y., Ott, M., Goyal, N., Du, J., Joshi, M., Chen, D., . . . Stoyanov, V. (2019). Roberta: A robustly optimized BERT pretraining approach. CoRR, Retrieved from arXiv:1907.11692

[CR56] Louwerse, M. M. (2018). Knowing the meaning of a word by the linguistic and perceptual company it keeps. *Topics in cognitive science,**10*(3), 573–589. 29851286 10.1111/tops.12349

[CR57] Lupyan, G., & Winter, B. (2018). Language is more abstract than you think, or, why aren’t languages more iconic? *Philosophical Transactions of the Royal Society B: Biological Sciences,**373*(1752), 20170137. 10.1098/rstb.2017.0137 PMC6015821 29915005

[CR58] Lynott, D., Connell, L., Brysbaert, M., Brand, J., & Carney, J. (2019). The lancaster sensorimotor norms: multidimensional measures of perceptual and action strength for 40,000 english words. *Behavior Research Methods,* pp. 1–21. 10.3758/s13428-019-01316-z PMC7280349 31832879

[CR59] Majid, A. (2020). Human olfaction at the intersection of language, culture, and biology. *Trends in Cognitive Sciences*. 10.1016/j.tics.2020.11.005 33349546

[CR60] Marjieh, R., Sucholutsky, I., van Rijn, P., Jacoby, N., & Griffiths, T. L. (2024). Large language models predict human sensory judgments across six modalities. *Scientific Reports,**14*(1), 21445. 39271909 10.1038/s41598-024-72071-1 PMC11399123

[CR61] Martínez, G., Molero, J. D., González, S., Conde, J., Brysbaert, M., & Reviriego, P. (2024). Using large language models to estimate features of multi-word expressions: Concreteness, valence, arousal. *Behavior Research Methods,**57*(1), 5. 39633225 10.3758/s13428-024-02515-z

[CR62] Martínez, G., Conde, J., Reviriego, P., & Brysbaert, M. (2025a). Ai-generated estimates of familiarity, concreteness, valence, and arousal for over 100,000 spanish words. *Quarterly Journal of Experimental Psychology,**78*(10), 2272–2283. 10.1177/17470218241306694 39614682

[CR63] Martínez, G., Conde, J., Reviriego, P., & Brysbaert, M. (2025b). Simulating lexical decision times with large language models to supplement megastudies and crowdsourcing. *Behavior Research Methods,**57*(10), 294. 40987999 10.3758/s13428-025-02829-6

[CR64] Miceli, A., Wauthia, E., Lefebvre, L., Ris, L., & Simoes Loureiro, I. (2021). Perceptual and interoceptive strength norms for 270 french words. *Frontiers in Psychology,**12*, 2018. 10.3389/fpsyg.2021.667271 PMC8226098 34177725

[CR65] Muraki, E. J., Siddiqui, I. A., & Pexman, P. M. (2022). Quantifying children’s sensorimotor experience: Child body-object interaction ratings for 3359 english words. *Behavior Research Methods,**54*(6), 2864–2877. 35112287 10.3758/s13428-022-01798-4

[CR66] OpenAI (2024). *GPT-4o System Card*. https://openai.com/index/gpt-4o-system-card/.

[CR67] Pexman, P. M., Muraki, E., Sidhu, D. M., Siakaluk, P. D., & Yap, M. J. (2019). Quantifying sensorimotor experience: Body-object interaction ratings for more than 9,000 english words. *Behavior research methods,**51*(2), 453–466. 30484218 10.3758/s13428-018-1171-z

[CR68] Ponari, M., Norbury, C. F., & Vigliocco, G. (2020). The role of emotional valence in learning novel abstract concepts. *Developmental Psychology,**56*(10), 1855. 32700948 10.1037/dev0001091

[CR69] Pulvermüller, F. (1999). Words in the brain’s language. *Behavioral and brain sciences,**22*(2), 253–279. 11301524

[CR70] Pustejovsky, J. (1991). The generative lexicon. *Computational linguistics,**17*(4), 409–441.

[CR71] Rabagliati, H., Marcus, G. F., & Pylkkänen, L. (2010). Shifting senses in lexical semantic development. *Cognition,**117*(1), 17–37. 20638655 10.1016/j.cognition.2010.06.007 PMC2934859

[CR72] Rabagliati, H., Marcus, G. F., & Pylkkänen, L. (2011). Rules, radical pragmatics and restrictions on regular polysemy. *Journal of Semantics,**28*(4), 485–512.

[CR73] Rayner, K., Pacht, J.M., & Duffy, S.A. (1994). Effects of prior encounter and global discourse bias on the processing of lexically ambiguous words: Evidence from eye fixations. *Journal of memory and language*, *33*(4), 527–544, Retrieved from https://www.sciencedirect.com/science/article/abs/pii/S0749596X84710254

[CR74] Reijnierse, W. G., Burgers, C., Bolognesi, M., & Krennmayr, T. (2019). How polysemy affects concreteness ratings: the case of metaphor. *Cognitive science,**43*(8), e12779. 31446656 10.1111/cogs.12779 PMC6771986

[CR75] Rivière, P. D., & Trott, S. (2025). Start making sense (s): A developmental probe of attention specialization using lexical ambiguity arXiv:2511.21974 arXiv preprint.

[CR76] Riviere, P.D., Beatty-Martínez, A.L., & Trott, S. (2025). Evaluating contextualized representations of (Spanish) ambiguous words: A new lexical resource and empirical analysis. In L. Chiruzzo, A. Ritter, & L. Wang (Eds.), *Proceedings of the 2025 conference of the nations of the americas chapter of the association for computational linguistics: Human language technologies (volume 1: Long papers)* (pp. 8322–8338). Albuquerque, New Mexico: Association for Computational Linguistics. Retrieved from https://aclanthology.org/2025.naacl-long.422/

[CR77] Rodd, J.M., Gaskell, M.G., & Marslen-Wilson, W.D. (2004). Modelling the effects of semantic ambiguity in word recognition. *Cognitive science*, *28*(1), 89–104. Retrieved https://onlinelibrary.wiley.com/doi/abs/10.1207/s15516709cog2801_4

[CR78] Sanh, V., Debut, L., Chaumond, J., & Wolf, T. (2019). DistilBERT, a distilled version of BERT: smaller, faster, cheaper and lighter. In *Proceedings of thirty-third conference on neural information processing systems (neurips2019).* Retrieved from arxiv.org/pdf/1910.01108

[CR79] Schlechtweg, D., Tahmasebi, N., Hengchen, S., Dubossarsky, H., & McGillivray, B. (2021). DWUG: A large resource of diachronic word usage graphs in four languages. arXiv preprint arXiv:2104.08540

[CR80] Schuhmann, C., Beaumont, R., Vencu, R., Gordon, C.W., Wightman, R., Cherti, M., . . . Jitsev, J. (2022). LAION-5b: An open large-scale dataset for training next generation image-text models. In *36th conference on neural information processing systems datasets and benchmarks track*. Retrieved from https://openreview.net/forum?id=M3Y74vmsMcY

[CR81] Scott, G. G., Keitel, A., Becirspahic, M., Yao, B., & Sereno, S. C. (2019). The glasgow norms: Ratings of 5,500 words on nine scales. *Behavior research methods,**51*(3), 1258–1270. 30206797 10.3758/s13428-018-1099-3 PMC6538586

[CR82] Singh, A., Hu, R., Goswami, V., Couairon, G., Galuba, W., Rohrbach, M., & Kiela, D. (2022). Flava: A foundational language and vision alignment model. In *Proceedings of the ieee/cvf conference on computer vision and pattern recognition* (pp. 15638–15650).

[CR83] Speed, L. J., & Brybaert, M. (2021). Dutch sensory modality norms. *Behavior Research Methods,* pp. 1–13. 10.3758/s13428-021-01656-9 PMC9170652 34505998

[CR84] Speed, L. J., & Majid, A. (2020). Grounding language in the neglected senses of touch, taste, and smell. *Cognitive neuropsychology,**37*(5–6), 363–392. 31230566 10.1080/02643294.2019.1623188

[CR85] Srinivasan, M., & Rabagliati, H. (2015). How concepts and conventions structure the lexicon: Cross-linguistic evidence from polysemy. *Lingua,**157*, 124–152.

[CR86] Srinivasan, M., Berner, C., & Rabagliati, H. (2019). Children use polysemy to structure new word meanings. *Journal of Experimental Psychology: General,**148*(5), 926. 30010371 10.1037/xge0000454

[CR87] Su, W., Zhu, X., Cao, Y., Li, B., Lu, L., Wei, F., & Dai, J. (2020). *Vl-bert: Pre-training of generic visual-linguistic representations*.

[CR88] Tamari, R., Shani, C., Hope, T., Petruck, M.R.L., Abend, O., & Shahaf, D. (2020). Language (re)modelling: Towards embodied language understanding. In *Proceedings of the 58th annual meeting of the association for computational linguistics* (pp. 6268–6281). Online: Association for Computational Linguistics. Retrieved from https://aclanthology.org/2020.acl-main.559

[CR89] Thompson, B., & Lupyan, G. (2018). Automatic estimation of lexical concreteness in 77 languages. In *The 40th annual conference of the cognitive science society (cogsci 2018)* (pp. 1122–1127).

[CR90] Trott, S. (2024a). Can large language models help augment english psycholinguistic datasets? *Behavior Research Methods,**56*(6), 6082–6100. 38261264 10.3758/s13428-024-02337-z PMC11335796

[CR91] Trott, S. (2024b). Large language models and the wisdom of small crowds. *Open Mind,**8*, 723–738. 38828431 10.1162/opmi_a_00144 PMC11142632

[CR92] Trott, S., & Bergen, B. (2020). Why do human languages have homophones? *Cognition,**205*, 104449. 32947137 10.1016/j.cognition.2020.104449

[CR93] Trott, S., & Bergen, B. (2021). RAW-C: Relatedness of ambiguous words in context (a new lexical resource for English). In *Proceedings of the 59th annual meeting of the association for computational linguistics and the 11th international joint conference on natural language processing (volume 1: Long papers)* (pp. 7077–7087). Online: Association for Computational Linguistics. Retrieved from https://aclanthology.org/2021.acl-long.550

[CR94] Trott, S., & Bergen, B. (2023). Word meaning is both categorical and continuous. *Psychological Review,**130*(5), 1239. 36892900 10.1037/rev0000420

[CR95] Trott, S., Torrent, T.T., Chang, N., & Schneider, N. (2020). (Re)construing meaning in NLP. In *Proceedings of the 58th annual meeting of the association for computational linguistics* (pp. 5170–5184). Online: Association for Computational Linguistics. Retrieved from https://aclanthology.org/2020.acl-main.462

[CR96] Trott, S., Jones, C., Chang, T., Michaelov, J., & Bergen, B. (2023). Do large language models know what humans know? *Cognitive Science,**47*(7), e13309. 37401923 10.1111/cogs.13309

[CR97] Turton, J., Vinson, D., & Smith, R. (2020). Extrapolating binder style word embeddings to new words. In *Proceedings of the second workshop on linguistic and neurocognitive resources* (pp. 1–8). Marseille, France: European Language Resources Association. Retrieved from https://aclanthology.org/2020.lincr-1.1

[CR98] Utsumi, A. (2020). Exploring what is encoded in distributional word vectors: A neurobiologically motivated analysis. *Cognitive Science,**44*(6), e12844. 32458523 10.1111/cogs.12844

[CR99] Vinaya, H., Trott, S., Pecher, D., Zeelenberg, R., & Coulson, S. (2025). Words and worlds both: Dynamic effects of distributional and sensorimotor information in semantic processing. *Open Mind,**9*, 2149–2174. 41497522 10.1162/OPMI.a.316 PMC12768550

[CR100] Wan, D., Kedzie, C., Ladhak, F., Carpuat, M., & McKeown, K. (2020a). Incorporating terminology constraints in automatic post-editing. In *Proceedings of the 5th conference on machine translation* (pp. 1193–1204). Online: Association for Computational Linguistics. Retrieved from https://aclanthology.org/2020.wmt-1.141

[CR101] Wan, M., Xing, B., Su, Q., Liu, P., & Huang, C.- R. (2020). Sensorimotor enhanced neural network for metaphor detection. In *Proceedings of the 34th pacific asia conference on language, information and computation* (pp. 312–317). Hanoi, Vietnam: Association for Computational Linguistics. Retrieved from https://aclanthology.org/2020.paclic-1.36

[CR102] Wiley, J., George, T., & Rayner, K. (2018). Baseball fans don’t like lumpy batters: Influence of domain knowledge on the access of subordinate meanings. *Quarterly Journal of Experimental Psychology,**71*(1), 93–102. 10.1080/17470218.2016.1251470 27767384

[CR103] Wingfield, C., & Connell, L. (2021). Sensorimotor distance: A fully grounded measure of semantic similarity for 800 million concept pairs. 10.3758/s13428-022-01965-7 PMC10615916 36131199

[CR104] Wingfield, C., & Connell, L. (2023). Sensorimotor distance: A grounded measure of semantic similarity for 800 million concept pairs. *Behavior Research Methods,**55*(7), 3416–3432. 36131199 10.3758/s13428-022-01965-7 PMC10615916

[CR105] Winter, B., & Bergen, B. (2012). Language comprehenders represent object distance both visually and auditorily. *Language and Cognition,**4*(1), 1–16.

[CR106] Winter, B., & Srinivasan, M. (2022). Why is semantic change asymmetric? the role of concreteness and word frequency and metaphor and metonymy. *Metaphor and Symbol,**37*(1), 39–54.

[CR107] Winter, B., Lupyan, G., Perry, L. K., Dingemanse, M., & Perlman, M. (2024). Iconicity ratings for 14,000+ english words. *Behavior research methods,**56*(3), 1640–1655. 37081237 10.3758/s13428-023-02112-6

[CR108] Wu, M., Conde, J., Reviriego, P., & Brysbaert, M. (2026). How does fine-tuning improve sensorimotor representations in large language models? arXiv:2603.03313 arXiv preprint.

[CR109] Xu, Q., Peng, Y., Nastase, S. A., Chodorow, M., Wu, M., & Li, P. (2025). Large language models without grounding recover non-sensorimotor but not sensorimotor features of human concepts. *Nature human behaviour,**9*(9), 1871–1886. 40468013 10.1038/s41562-025-02203-8 PMC12454144

[CR110] Yee, E., & Thompson-Schill, S.L. (2016). Putting concepts into context. *Psychonomic bulletin & review*, *23*(4), 1015–1027, Retrieved from https://link.springer.com/article/10.3758/s13423-015-0948-7 10.3758/s13423-015-0948-7 PMC4975661 27282993

[CR111] Zellers, R., Lu, X., Hessel, J., Yu, Y., Park, J. S., Cao, J., & Choi, Y. (2021). Merlot: Multimodal neural script knowledge models arXiv:2106.02636 arXiv preprint.

